# Age-related changes in reach-to-grasp movements with partial visual occlusion

**DOI:** 10.1371/journal.pone.0221320

**Published:** 2019-08-28

**Authors:** Nuttakarn Runnarong, Jarugool Tretriluxana, Watinee Waiyasil, Preeyanuch Sittisupapong, Suradej Tretriluxana

**Affiliations:** 1 Faculty of Physical Therapy, Srinakharinwirot University, Nakhon Nayok, Thailand; 2 Motor Control and Neural Plasticity Laboratory, Faculty of Physical Therapy, Mahidol University, Nakhon Pathom, Thailand; 3 Faculty of Engineering, King Mongkut’s Institute of Technology Ladkrabang, Bangkok, Thailand; University of Exeter, UNITED KINGDOM

## Abstract

This study investigated the influence of age and visual occlusion on fast reach-to-grasp movements. The effect of visual occlusion on reach-to-grasp movement was examined using a task that heavily relies on feed-forward control. Three groups of healthy adults aged 22, 49 and 65 on average performed fast reach-to-grasp movements with full visual and partial visual occlusion conditions of the hand during the initial part of movement. Regarding the effect of age, the all parameters of reach-to-grasp movement were deteriorated with age, except relative time to maximum velocity and spatial coordination. Regarding the effect of visual condition, participants reached with prolonged movement time, lower peak velocity, and later occurrences of peak velocity and peak aperture, as well as decrease in spatial coordination. Regarding the effect of age on visual condition, visual occlusion resulted in a longer movement time and delayed time to maximum velocity in middle-aged and older groups compared to full vision, but the difference was not observed in the younger groups. Conclusion: Reach-to-grasp performance deteriorated with age and the performance was affected when vision of the hand at initial movement was occluded. Overall, movement performance in middle-aged and older adults was affected by visual occlusion, whereas it was unaffected in younger adults. The results indicate that visual feedback of the hand at initial movement is important to control reach-to-grasp movement of middle-aged and older adults during real tasks.

## Introduction

Activities of daily living are primarily upper extremity tasks, and many of them involve reach-to-grasp movements [1]. Reach-to-grasp (RTG) is a complex motor skill that requires integration of planning and execution of reaching (transport) and grasping movements, which are termed higher-order control systems [2]. Performance of RTG movement changes significantly with age. Older adults demonstrated longer movement duration and change in relative time to peak velocity and peak aperture [3–5]. Moreover, coordination of transport and grasp components showed a subtle decrease, but was more evident in high-precision tasks [3, 6]. Older adults also demonstrated appropriate kinematic modifications showing that they can adopt strategies to compensate for deterioration [3]. As previously known, physiological change leads to sensory, motor, and neural capability change gradually occurring in middle age, which subsequently declines until quality of life of elderly individuals is affected [7]. Currently, evidence shows a decline of manual dexterity and a decrease of grip force control starting to occur in middle-aged adults [4, 8]. However, research studies focusing on RTG control in middle-aged adults is relatively limited.

With regards to the effect of visual information, recent evidence showed that vision of hand and object in early phase of RTG movement is crucial for grasp control [9]. Viewing the object early during grasping has more influence on program and control grasping than the viewing of the hand. A previous study reported that occlusion of both hand and object led to a larger maximum aperture, whereas early occlusion of the hand did not influence the maximum aperture [9]. In line with the previous study, Connolly and Goodale (1999) also found that occlusion of the moving hand had no effect on maximum aperture but had clear effects on the kinematics of reach [10]. Moreover, vision occlusion of the moving hand at the initial part of movement affected RTG coordination and grasp pre-shaping, which implied the role of feed-forward control in this movement [11]. Although, these studies revealed the role of different visual cues influencing RTG control, most of these studies have been conducted in younger adults [9, 10]. Knowledge on the role of visual cues of hand movement on the motor strategy in advanced age, particularly at the beginning of the physiological change in middle age, is limited. To the best of our knowledge, the effect of visual occlusion of hand movement in aging has been reported in one previous study [12]. The result of the study showed that vision occlusion of the hand affected the kinematics of RTG movement, but the magnitude of the effect was larger in older adults compared with younger adults. The study suggested that older adults were more reliant on visual feedback than younger adults when visualization of the reaching hand was removed.

In addition to the visual feedback, the change and slowing of feed-forward in older adults has also been well reported in several goal-directed tasks. For object-lifting tasks, evidence showed that older adults tended to rely more on the internal model or feed-forward control compared with younger adults to compensate for the less afferent feedback [13]. With regard to RTG task, investigating the RTG kinematics and coordination in high degree of difficulty using obstacle avoidance in older adults including participants with stroke or Parkinson’s disease, RTG coordination deficits have been detected [14–16]. Using fast RTG movement under visual occlusion results in minimizing the use of visual feedback from the arm and hand and maximize the use of feed-forward control [11]. During perform the fast movement, visuomotor system was limited time to use vision and online control to improve the RTG performance. This experimental paradigm was used to investigate differences of anticipatory planning between two hands. Superior in anticipatory planning between hands was found when performed the RTG movement under visual occlusion, but not under full vision [11]. Therefore, visual occlusion or full vision would have influence on anticipatory planning in RTG movement. The previous findings supported that the central nervous system heavily relies on the use of feed-forward control when using vision occlusion of the arm and hand at the initial part of movement [11]. However, whether the ability to respond to the task, which heavily relies on the use of feed-forward control, i.e. when there is early visual occlusion of hand movement, is different between the age groups is still unknown. With increasing age, the change in movement strategy allows older adults to perform the task without error. Older adults showed more rigid movement pattern to unanticipated RTG movement [6] and more conservative strategy in natural RTG tasks compared with younger adults [17]. These strategies may be adopted to optimize movement time and energy expenditure in daily tasks with high precision. Several studies have reported RTG strategy changes in older adults [6, 17]; however, studies investigating whether middle-aged adults might adopt strategies in RTG control to compensate for age-related passive degenerative processes are scarce. Only a few studies have assessed reach-to-grasp performance across a life span and investigated the effect of visual occlusion in virtual environments. Different movement strategies among younger, middle-aged and older groups were reported when performing precision grasp [18, 19]. Younger adults have the ability to utilize visual information differently compared to middle-aged and older adults [18]. However, performing the task in a virtual environment might utilize the movement kinematics differently from the real workplace similar to our study [5, 18]. In addition, there is evidence that brain activity is altered between experiences in virtual reality and real world [20, 21]. Knowledge on the different motor strategies in advancing age may improve understanding regarding neural control on behavioral deterioration [22, 23]. Therefore, the first and the second aim of this study were to investigate the effect of age and to determine the effect of visual occlusion on reach-to-grasp movements. The third aim was to determine whether reach-to-grasp performance with different visual conditions is differentially affected by age. We hypothesized that RTG performance would be deteriorated in middle-aged and older adults. We also hypothesized that RTG performance would be deteriorated when vision of the hand was occluded at the initial movement. Kinematics of RTG movement in reach and grasp components, as well as its coordination would be weakened in aging groups and in a visual occlusion condition. In addition, we hypothesized that performance of RTG movement with different visual conditions would vary depending on age.

## Materials and methods

### Participants

Thirty-six healthy adults (21 women, 5 men) age ranged between 21 to 73 years participated in the study. Participants who met inclusion and exclusion criteria were divided into three groups including younger (age, 21–24 years), middle-aged (age, 41–55 years), and older adult groups (age, 61–73). There are twelve participants in each age group. All participants were right-handed as determined by the Edinburg Handedness Inventory. The participants met the following criteria: ability to understand the task instructions with score > 24 by Thai Mini Mental Status, no hearing and visual problems not correctable with lens, and no neurological problems. The participants were excluded if the following criteria were met: blood pressure >140/90 mmHg, presence of joint limitation or pain at the upper extremity. The participants were recruited from a local community. The Ethics committee of Srinakharinwirot University approved the study (PTPT 2017–005). All participants obtained written informed consent before the study was conducted. The individual in this manuscript has given written informed consent to publish these case details.

### Experimental setup

**Fig 1:**
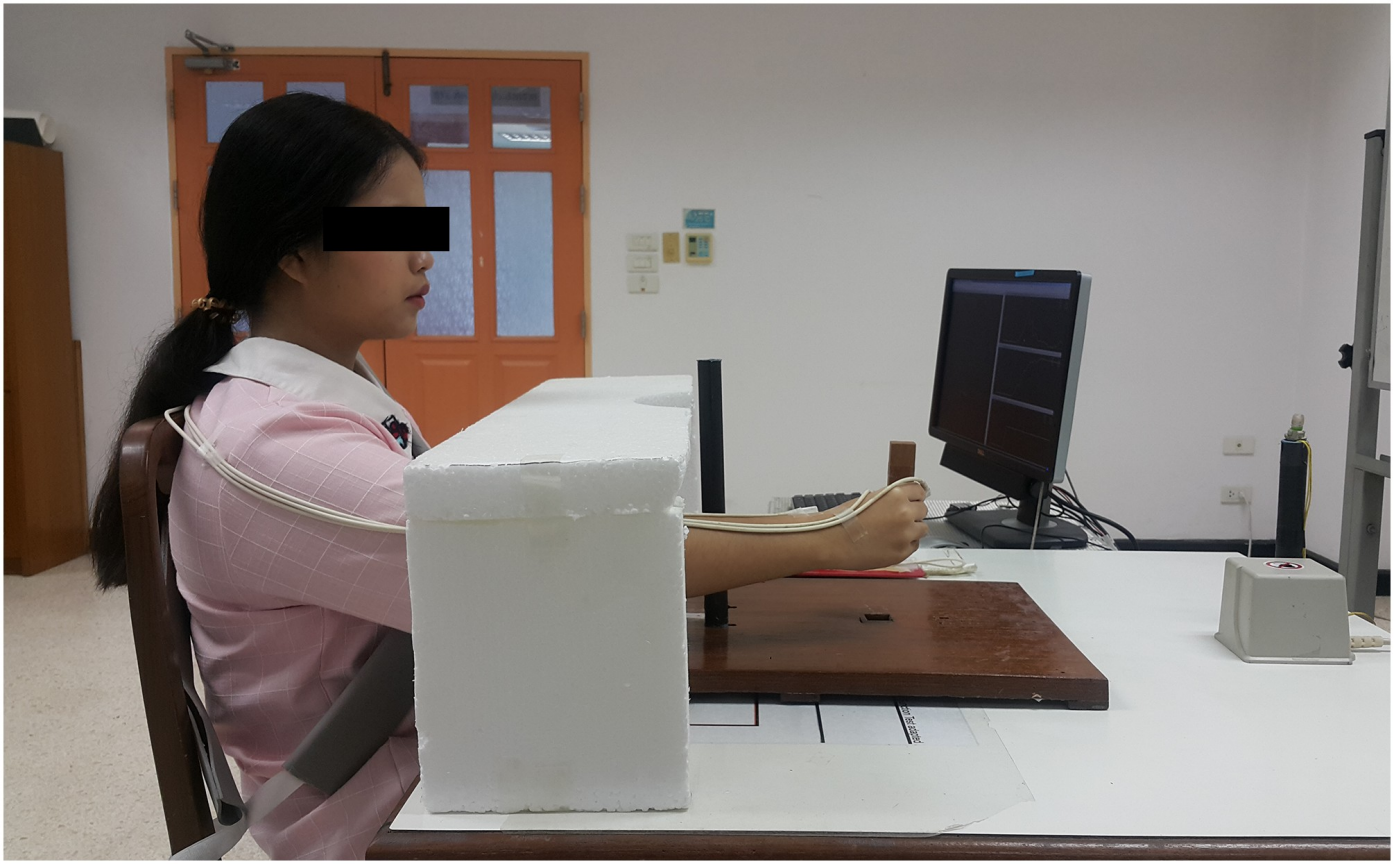
Experimental setup of the RTG task. Reach-to-grasp task setup in visual occlusion condition (A). Position of the barrier and the target object from the top view (B).

The experimental setup of the RTG task is shown in Fig 1. All participants were seated comfortably in front of the experimental table with the trunk secured by a belt and the feet supported on the floor. A square object (2.5 cm in width and 10 cm in height) was located 30 cm along the midline of the trunk from the area where the hand was placed on the start switch. The object was marked at the center to designate where the fingers should be contacted. The barrier was cylindrical (2 cm in diameter and 30 cm in height) located 15 cm in front of the start switch and 2.5 cm away from the midline between the start switch to the target object. The barrier was placed at the right to force a curved hand path of the reach [11]. Performing the movement with the barrier in place serves to increase the task difficulty by challenging the movement planning and transport-grasp coordination elements of a RTG movement [11, 24].

In addition, RTG movement was performed with the right hand with full vision and visual occlusion conditions. Six participants in each group started with a full vision condition, and the other six participants began with a visual occlusion condition to counterbalance between different visual conditions. For the visual occlusion condition, the foamed shield was placed between the starting switch and the barrier, such that it occluded vision of the arm and hand at the initial part of movement and later allowed vision at the end of the movement (Fig 1). Under vision occlusion of the arm and hand at movement initiation, the participant had to minimize the use of visual feedback from the arm and heavily rely on the use of feed-forward control during the movement initiation [11].

Before data collection, the experimenter demonstrated the task. Subsequently, the participants were allowed to practice with full vision one time at their preferred speed and three times at a faster speed. This practice session allowed the participants to be aware of the fast speed which was required during the actual testing. The foamed shield was placed between the starting switch and barrier before data recording.

### RTG task

Before each trial, the participants were asked to place their right hand at the start switch with the thumb and index fingertips slightly touching each other at the tips. The hand and forearm of the testing limb were placed on the table as a starting position. The other hand was placed on the lap during experiment testing. Light-emitting diode (LED) was used as a signal to initiate movement. The LED light was positioned behind the object and 20 cm above the table surface. All participants were instructed to move immediately after the LED emitted light. The instruction was “upon LED activation, reach as fast as possible without collision of the barrier, grasp the object with the thumb and index fingers, and lift it off a few centimeters from the table”. Fifteen completed trials in each visual condition were averaged and used in the data analysis. Moreover, the participants were allowed to take a 1-min or several-minute break to avoid fatigue.

### Data acquisition and analysis

The kinematic data of RTG movement was recorded using an electromagnetic tracking motion system (MotionMonitor™, Innsport, Chicago, IL, USA), with six degree-of-freedom Mini-Bird sensors (Ascension Technologies, Burlington, VT, USA). Three sensors were attached on the upper extremity. The wrist sensor was attached on the styloid process of the wrist. The other two sensors were attached on the nail beds of the thumb and index fingers. All kinematic data was filtered using a zero-lag Butterworth low-pass filter with a cut-off frequency of 20 Hz. The sampling rate for the three sensors was 100 Hz. The data was analyzed using the Matlab Signal Processing Toolbox.

**Fig 2:**
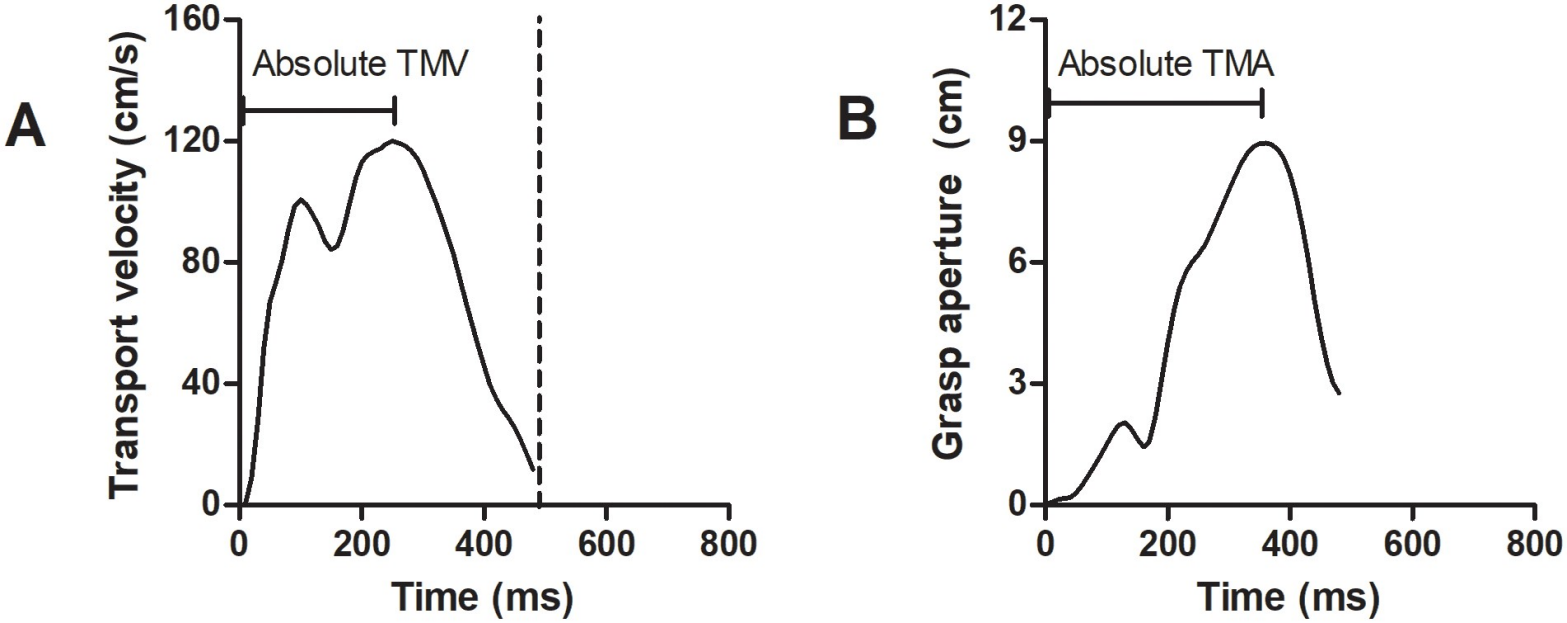
Dependent variables. Transport velocity (A) and grasp aperture profiles (B).

Kinematic characteristics related to the transport component and grasp component were analyzed. For the transport component, three-dimensional displacement was derived from the wrist sensor position. Tangential velocity of transport was calculated from the displacement data of wrist sensor. The displacement data from x, y, and z coordinates of the wrist sensor were differentiated to tangential velocity using a finite-different technique [25]. For grasp component, three-dimensional displacement was derived from thumb and index finger sensors. The grasp aperture size was calculated from the resultant distance between the thumb and index finger sensors. The higher peak of grasp apertures after passing the barrier was used to show grasp pre-shaping. The dependent variables in this study included 1) total movement time (TMT): the time between movement initiation (the time before the release of thumb and index finger from the start switch at which the tangential transport velocity was > 0.4 cm/s) and movement termination (indicated by a tangential transport velocity reaching the lowest velocity before the object was lifted off); 2) Transport component: maximum transport velocity (MV) was defined as the maximum value of transport velocity; absolute time of maximum transport velocity (TMV) was defined as the duration from the movement initiation to the maximum transport velocity (Fig 2); relative time of maximum transport velocity (%TMV) indicated acceleration time of the transport component which was defined as the absolute time of maximum transport velocity that was normalized by total movement time of each trial; deceleration time (DT) was defined as the duration from the peak of transport velocity to the movement termination. TMT, MV, TMT, %TMV and DT were based on the wrist sensor; 3) Grasp component: maximum aperture (MA) was defined as maximum resultant distance between the thumb and index finger sensors of the maximum peak of grasp aperture; absolute time of maximum aperture (TMA) was defined as the duration from movement initiation to the maximum grasp aperture (Fig 2); relative time of maximum aperture (%TMA) which was defined as the absolute time of maximum aperture that was normalized by total movement time of each trial. MA, TMA and %TMA were based on the thumb and index finger sensors; and 4) RTG coordination was measured by a cross correlation analysis between transport velocity and aperture size. The coordination was expressed by cross-correlation coefficient (r) as a function of time lag (T). Spatial coordination was derived from the correlation coefficient at the most similar pattern between the transport velocity and grasp aperture trajectories, called maximum correlation coefficient (r_max_). Temporal coordination, called maximum time lag (T_max_), was quantified by the time used to shift the transport velocity trajectory relative to the grasp aperture trajectory until the r_max_ was detected [16]. Strong coordination was evaluated from the higher r_max_ and lower T_max_.

### Statistical approach

For each participant, the mean values for each of the dependent measures were calculated for each age group (older/middle/younger) × visual conditions (full vision/visual occlusion). An analysis of variance (two-way mixed ANOVA) was conducted with age group as the between-subjects factor (older/middle/younger) and visual conditions (full vision/visual occlusion) as within-subject factors. Before performing ANOVA, normal distribution of the data was verified. Post hoc comparisons were performed using Bonferroni. Pairwise comparisons with Bonferroni correction were used to interpret significant main effects. All significant interactions were further explored by using one-way ANOVA to examine age group differences at each visual condition, and paired sample t-test to examine differences between visual conditions at each age group. The level of statistically significant difference for all analyses was set at p < 0.05.

## Results

Thirty-six healthy adults participated in the study. The mean age of the three age groups were 22.41 years, 49.16 years, and 65.5 years for young, middle-aged, and older adults, respectively. The characteristics of the participants based on the different age groups are summarized in Table 1.

**Table 1:** Participant characteristics.

Group	N	Age (years) Mean ± SD	Age range (years)	Sex Female/male
Young adult	12	22.41±0.95	21–24	7/5
Middle-aged adult	12	49.16±5.07	41–55	6/6
Older adult	12	65.50±4.01	61–73	8/4

### Effect of age groups and visual conditions

**Fig 3:**
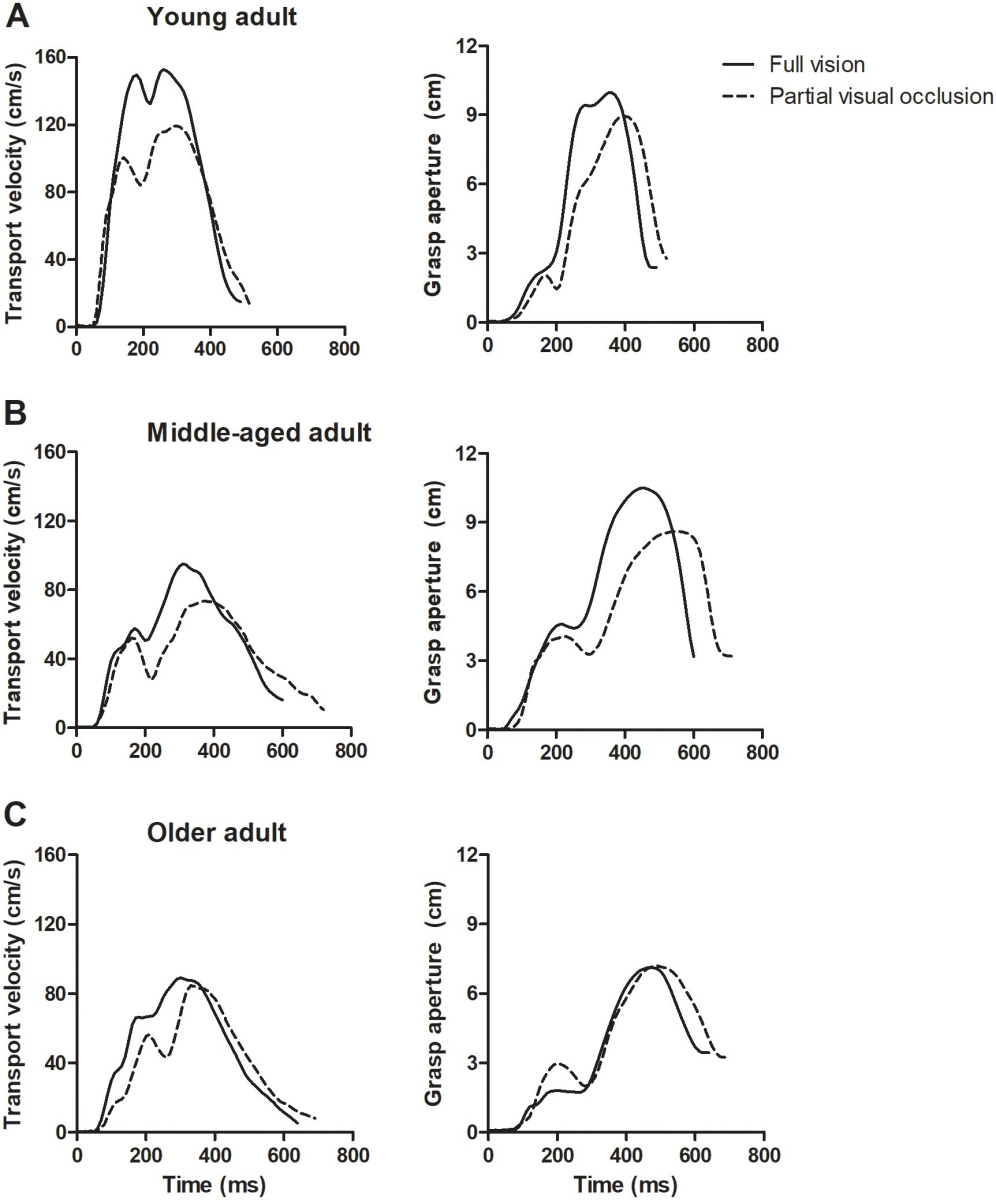
Transport velocity and grasp aperture profiles. Sample transport velocity and grasp aperture profiles of 2 visual conditions (*solid line* full vision, *dashed line* partial visual occlusion) for younger adults (A), middle-aged adults (B) and older adults (C).

Sample transport velocity and grasp aperture profiles of full visual and partial visual occlusion conditions for a representative younger, middle-aged and older adults are shown in Fig 3. Compared to the younger group, aging groups exhibited longer movement time when reach-to-grasp with full vision and partial visual occlusion.

The means and standard deviations for all dependent variables are reported in Table 2. A significant main effect of age was found for all dependent variables, except %TMV and r_max_. A significant main effect of visual condition was found for TMT, MV, TMV, TMA, %TMV, %TMA and r_max_. Significant interactions between age and visual condition was found for TMT and TMV. These results will be reported in detail.

**Table 2:** Mean and standard deviations for all dependent variables.

Variables	Age group			Visual condition	
	Younger	Middle-aged	Older	Full vision	Visual occlusion
TMT (ms)	418.61 (51.54)	598.41 (91.93)	632.69 (116.21)	528.63 (119.56)	571.18 (138.14)
MV (cm/s)	141.25 (23.79)	96.39 (11.20)	95.95 (13.24)	117.33 (28.10)	105.067 (25.13)
TMV (ms)	153.58 (35.21)	258.25 (59.98)	254.63 (69.39)	206.29 (62.22)	238.01 (82.43)
TMA (ms)	270.75 (37.54)	432.02 (67.85)	438.22 (99.58)	359.57 (100.21)	401.09 (109.11)
DT (ms)	265.02 (50.61)	340.16 (61.67)	378.05 (76.42)	322.33 (78.05)	333.16 (80.04)
%TMV	37.00 (8.47)	43.14 (6.93)	40.30 (6.75)	38.99 (7.13)	41.30 (8.25)
%TMA	64.91 (5.11)	72.35 (3.95)	69.26 (6.90)	67.80 (7.50)	69.88 (4.39)
MA (cm)	10.17 (1.28)	8.78 (1.52)	8.15 (1.59)	8.99 (1.65)	9.08 (1.73)
T_max_ (ms)	105.75 (34.29)	162.86 (25.92)	178.00 (49.32)	154.48 (49.00)	143.25 (48.35)
r_max_	0.87 (0.06)	0.84 (0.07)	0.84 (0.07)	0.86 (0.06)	0.84 (0.07)

**Fig 4:**
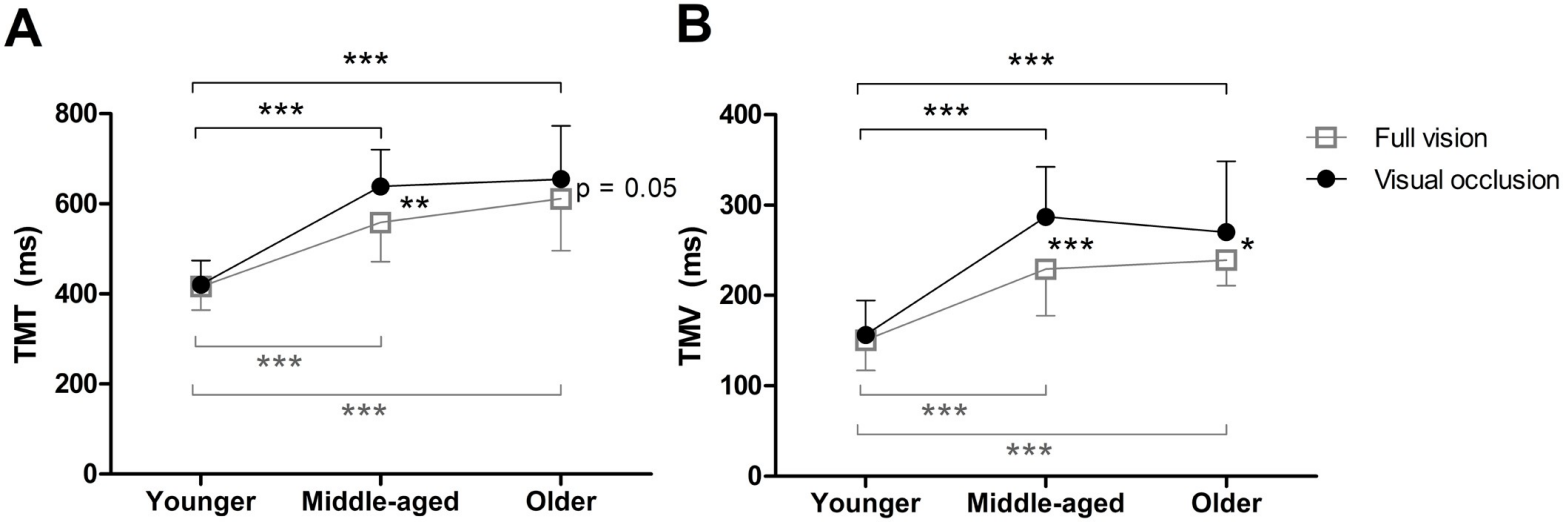
Illustration of age-by-visual condition interaction with post-hoc analysis. Total movement time (TMT) (A), time to maximum velocity (TMV) (B). *P-values* were shown for significance between the age groups and between the visual conditions with *p < 0.05, **p = 0.002, ***p ≤ 0.001. Analyzing the difference between visual conditions in each age group using paired sample t-test; analyzing the difference between age groups in each visual condition using one-way ANOVA. Error bars represented standard deviation.

Total movement time (TMT). There was a main effect of age (F_2, 33_ = 23.45, p < 0.0001) driven by significant differences between the young group and both the other groups (younger vs middle-aged, p < 0.0001; younger vs older, p < 0.0001). There was no significant difference between middle-aged and older groups (p > 0.05). There was also a main effect of visual condition (F_1, 33_ = 15.46, p < 0.0001) with movements taking longer in the visual occlusion condition compared to full vision (p < 0.001). A significant interaction between age and visual condition emerged (F_2, 33_ = 4.14, p < 0.05). Post hoc test using one-way ANOVA showed middle-aged and older groups had a longer movement time compared to the younger group in both full vision and visual occlusion conditions (full vision: middle-aged (558.33±86.47 ms) vs younger (416.66±52.39 ms), p = 0.001; older (610.88±115.07 ms) vs younger, p < 0.0001 and visual occlusion: middle-aged (638.50±81.79 ms) vs younger (420.55±52.93 ms), p < 0.0001; older (654.50±118.15 ms) vs younger, p < 0.0001) (Fig 4A). Post hoc test using paired sample t-test revealed that visual occlusion made a longer movement time in middle-aged (p = 0.002) and tended to be a longer movement time, but it is not statistically significant in older age groups (p = 0.05) when compared to full vision. This statistic might reach significance if the sample size was increased, which needs to be further investigated. However, this difference was not observed in the younger group (p = 0.803) (Fig 4A).

Maximum velocity (MV). There was a main effect of age (F_2, 33_ = 37.96, p < 0.0001) with significant higher MV between the younger group and both the other groups (younger vs middle-aged, p < 0.0001; young vs older, p < 0.0001). There was no significant difference between middle-aged and older groups (p > 0.05). There was also main effect of visual condition (F_1, 33_ = 29.92, p < 0.0001) with significantly lower MV for the visual occlusion compared to the full condition (p < 0.0001). There was no significant interaction between group and visual condition.

#### Absolute timing

Time to maximum velocity (TMV) and Time to maximum aperture (TMA). There was a main effect of age (TMV, F_2, 33_ = 16.00, p < 0.0001; TMA, F_2, 33_ = 26.25, p < 0.0001) with significantly shorter TMV and TMA between the younger group and both the other groups (TMV: younger vs middle-aged, p < 0.0001; younger vs older, p < 0.0001) and TMA: younger vs middle-aged, p < 0.0001; younger vs older, p < 0.0001). There was no significant difference between middle-aged and older groups for both absolute timings (p > 0.05). There was also a main effect of visual condition (TMV, F_1, 33_ = 28.28, p < 0.0001; TMA, F_1, 33_ = 17.48, p < 0.0001) with TMV and TMA that occurred significantly later in the visual occlusion condition compared to full vision (TMV, p < 0.0001; TMA, p < 0.0001).

There was no significant interaction between age and visual condition on TMA. However, there was significant interaction between age and visual condition on TMV (F_2, 33_ = 6.365, p = 0.005). Post hoc test using one-way ANOVA showed middle-aged and older age groups had a prolonged TMV compared to the younger group in both visual conditions (full vision: middle-aged (229.16±51.49 ms) vs younger (150.55±33.53 ms), p = 0.001; older (239.16± 28.35 ms) vs younger, p < 0.0001 and visual occlusion: middle-aged (287.33±55.01 ms) vs younger (156.61±38.05 ms), p < 0.0001; older (270.11±78.35 ms) vs younger, p < 0.0001) (Fig 4B). Post hoc test using paired sample t-test revealed that visual occlusion made a prolonged TMV in middle-aged (p < 0.0001) and older groups (p = 0.04) compared to full vision. The younger group did not show any difference between visual conditions (Fig 4B).

Deceleration time (DT). The age effect was statistically significant (F_2, 33_ = 11.07, p < 0.0001). Deceleration time was longer for middle-aged and older groups compared with the younger group (younger vs middle-aged, p < 0.05; younger vs older, p < 0.0001). There was no significant difference between middle-aged and older groups (p > 0.05). However, the visual condition effect did not reach any significance (F_1, 33_ = 1.74, p > 0.05). There was no significant interaction between age and visual condition.

#### Relative timing

Relative time to maximum velocity (%TMV). There was no main effect of age (%TMV, F_2, 33_ = 2.29, p > 0.05). However, there was a main effect of visual condition (%TMV, F_1, 33_ = 7.264, p < 0.05), with %TMV occurring later in visual occlusion compared to a full visual condition (p < 0.05). There was no significant interaction between age and visual condition.

Relative time to maximum aperture (%TMA). There was a main effect of age (%TMA, F_2, 33_ = 7.79, p = 0.002), with %TMA occurring significantly later only in the middle-aged group compared to the younger group (p ≤ 0.001). There was also a main effect of visual condition (%TMA, F_1, 33_ = 4.967, p < 0.05), with %TMA occurring later in visual occlusion compared to a full visual condition (p < 0.05). There was no significant interaction between age and visual condition.

#### Maximum aperture

Maximum aperture (MA). There was a main effect of age (F_2, 33_ = 6.50, p = 0.004) with a significant smaller grasp aperture only in the older group compared to the younger group (p = 0.004). There was no main effect of visual condition (MA, F_1, 33_ = 0.21, p > 0.05). There was no significant interaction between age and visual condition.

#### RTG coordination

Maximum time lag (T_max_). There was a main effect of age (T_max_, F_2, 33_ = 15.79, p < 0.0001), with T_max_ being significantly longer in the older and the middle-aged group compared to the younger group (older vs younger, p < 0.0001; middle-aged vs younger, p ≤ 0.001). There were no significant differences between middle-aged and older groups (p > 0.05). Whereas, there was no main effect of visual condition (F2, 33 = 0.70, p > 0.05). There was no significant interaction between age and visual condition.

Maximum correlation coefficient (r_max_). There was no main effect of age, but there was a main effect of visual condition (F_1, 33_ = 4.55, p < 0.05), with a significantly lower r_max_ in visual occlusion condition compared to full vision (p < 0.05). There was no significant interaction between age and visual condition.

**Fig 5:**
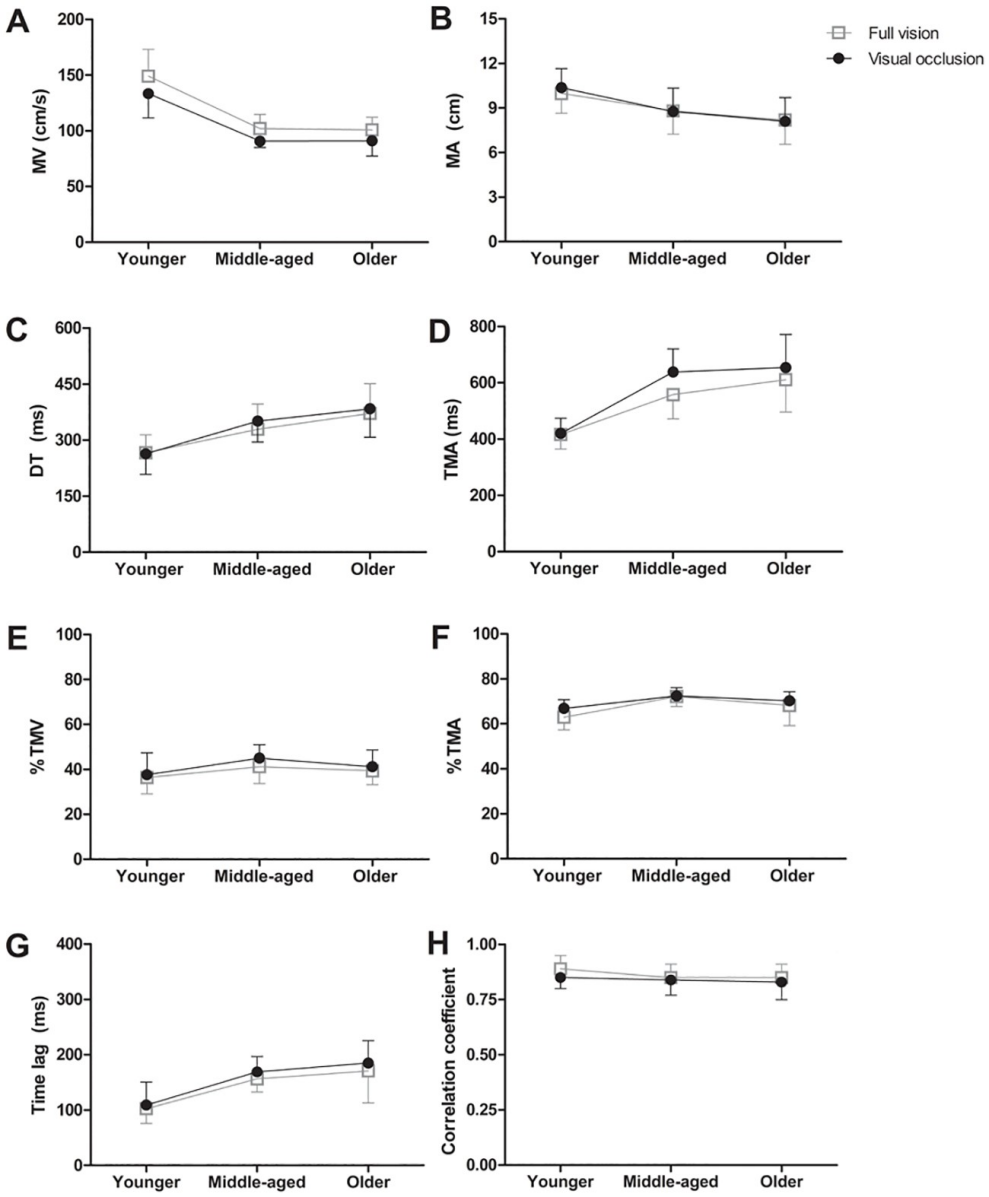
(A-H). Illustration of dependent variables in full-vision and visual occlusion conditions in each age group. Maximum transport velocity (MV) (A), maximum aperture (MA) (B), deceleration time (DT) (C), absolute time of maximum aperture (TMA) (D), relative time of maximum transport velocity (%TMV) (E), relative time of maximum aperture (%TMA) (F), maximum time lag (T_max_) (G), maximum correlation coefficient (r_max_) (H). No significant interaction of age-by-visual condition. Error bars represented standard deviation.

Figs 4 and 5 show the means and SDs between full-vision and visual occlusion conditions of all kinematic parameters.

## Discussion

The aim of the study was to determine the effect of age and visual occlusion when performing the RTG task with visual occlusion of the initial hand movement. Furthermore, we also determined whether the RTG performance with different visual occlusion conditions was differentially affected by age. Our findings demonstrated that RTG performances were deteriorated with age and were decreased when vision was occluded. Moreover, overall movement performances were deteriorated in aging groups when vision was occluded, while there was no evident deterioration in younger adults. This is the first study to demonstrate the effect of visual occlusion when performing RTG movements in a real life task in regards to the participants’ ages.

### Effect of aging

All parameters in our study were deteriorated with age except relative time to maximum velocity and spatial coordination. Relative time to maximum velocity was not changed in aging groups, possibly due to a prolonged movement time and later occurrence of peak velocity for preserving the consistent ratio of duration in transport component relative to total movement time [3, 14]. Aging groups that preserved a spatial coordination are in line with the previous study which reported that movement pattern was similar between younger and older adults [3]. It is possible that participants in aging groups need extended time to coordinate transport velocity and grasp aperture in order to preserve the similarity of the pattern of movements.

### Effect of visual condition

The occluded vision of the arm and hand at the beginning of the movement deteriorated the RTG performance. Under visual occlusion, participants reached with prolonged overall movement performance, lower peak velocity, and later occurrences of timing and proportionally temporal setting of peak velocity and peak aperture, as well as disrupted spatial coordination. Our findings consistent with the previous studies that reported the role of vision of the hand during RTG movement [9, 10, 12]. Our evidence suggested that the visual information of the hand affected the RTG performance. The possible reason is the presence or absence of the visual information about the moving limb during the initial movement is important for the execution of the RTG movement.

### Effect of aging on visual condition

Our study demonstrated an interaction between age and visual condition in total movement time and time to peak velocity indicating that the effects of visual condition on RTG performance were different depending on age group. The results showed that overall movement performance in younger adults was unaffected by visual occlusion, whereas middle-aged and older adults took longer to complete movements when vision was occluded compared to full vision. The significant interaction of age and visual condition for overall movement time and time to peak velocity was further analyzed.

Older and middle-aged groups demonstrated a longer overall performance and later occurrence of peak velocity in both visual conditions, indicating a slow movement in aging groups. Slowing of RTG movement is a common compensatory strategy reflected in an alteration in movement control processes of aging [3, 22, 26]. Prolonged movement time in the middle-aged and older adults may result from reduced sensorimotor control, slow processing information, decreased motor planning, and deficits in neuromuscular control, leading to increased time to execute the task [8, 27–29]. The result demonstrated an increase in acceleration time in the aging groups, consistent with the findings of Grabowski and Mason (2014) [5]. Given the fact that we found no effect of age on %TMV it seems likely that the age differences found in TMV were driven by the increased movement time (TMT) in the older and middle-aged groups compared to the young [3].

The occlusion of the visual feedback of the hand at the initial movement did not affect overall movement performance in the younger age group. The results are in line with Fukui and Inui (2006) reported that younger adults can utilize visual information of the target while occluded vision of the hand at the initial movement [9]. It is possible that younger adults preserve their ability to process sensory information within limited visual feedback under visual occlusion [30]. Therefore, only visual information of the target is enough for the younger group to process the motor program to maintain overall performance. In contrast, aging groups, especially in the middle-aged group, spent longer movement time when vision of the hand at initial movement was occluded. It is possible that visual feedback of the hand at initial movement was important to control RTG movement. Aging groups are perhaps more reliant on visual feedback [12, 30]. Our findings of older adults are in line with the study of Coats et al 2011 [12] and reported that older groups relied more on visual feedback than the younger age groups when vision of the hand was occluded. For the middle-aged group, our results were consistent with the previous studies of reaching movement findings that young adults use visual information more efficiently than middle-aged adults, possible due to impaired ability to monitor online movement through visual feedback in middle-aged groups [28, 31]. Further investigation is required to clarify the possible reason as to why the middle-aged group were more affected than older adults when vision was occluded. In conclusion, our findings support that aging groups, especially middle-aged, tend to rely more on a visual feedback control when vision of the hand was occluded. Recently, Grabowski and Mason (2012 and 2019) [18, 32] investigated RTG performance in virtual reality and reported conflicting results with our findings. Previous studies revealed that middle-aged participants tend to use full visual feedback of the hand to improve RTG performance, while older participants have a tendency to rely more on a feed-forward strategy when vision was occluded [18]. In addition, the recent research reported visual occlusion using virtual reality did not affect movement time of RTG movement across different age groups [32]. In contrast, our results demonstrated that both middle-aged and older adults tended to be more reliant on visual feedback of the hand at the initial movement stage. These conflicting results may be due to the difference between a virtual reality and a real task. Different brain activity was observed and reported when comparing virtual reality to real world [20, 21]. Observation of real hand action activated a right posterior parietal cortex which related to motor processing. Whereas, observed virtual reality grasping action activated visual perceptual network which does not directly engage action representations [21]. Therefore, previous studies performed RTG movement in virtual environment which provided only visual information about the position of the thumb and index fingers during the movement. Visual information might not be sufficient enough to improve RTG performance [18, 32] when relating to our study. This may be the reason that the participants in middle-aged and older age groups in previous studies tended to rely more on feedforward planning in a virtual environment [32].

## Conclusions

Our study showed that kinematic RTG behavior had deteriorated with age. RTG performance also decreased when performing movement with visual occlusion compared to full vision. Moreover, our findings demonstrated that the effect of visual occlusion on RTG performance (in a real-world task) depended on the age group of the participant. Overall movement performance in middle-aged and older adults is affected by visual occlusion, whereas it is unaffected in younger adults. Thus implies that visual feedback of the hand in the initial movement was significant in controlling RTG movement in middle-aged and older adults, but not in younger adults.

The findings of our study may have broader implications for understanding the effects of visual occlusion in middle-aged and older adults. In addition, they provide significant information of different movement strategies among age groups, which may be beneficial for geriatric rehabilitation programs for different age ranges.

## Supporting information

S1 Table Mean values of all parameters for individual participant in full visual condition. Mean values of individual participant in full visual condition for younger, middle-aged and older groups.
(PDF)

S2 Table Mean values of all parameters for individual participant in visual occlusion condition. Mean values of individual participant in visual occlusion condition for younger, middle-aged and older groups.
(PDF)

S3 Table Raw data of individual participant in full visual condition. Raw data of individual participant in full visual condition for younger, middle-aged and older groups.
(PDF)

S4 Table Raw data of individual participant in visual occlusion condition. Raw data of individual participant in visual occlusion condition for younger, middle-aged and older groups.
(PDF)
