# Supplementary material for: Age-related changes in reach-to-grasp movements with partial visual occlusion
Source: PLoS One. 2019 Aug 28;14(8):e0221320. doi: 10.1371/journal.pone.0221320 (PMC6713340; doi:10.1371/journal.pone.0221320)
Supplement: S1 Table — Mean values of individual participant in full visual condition for younger, middle-aged and older groups. (PDF) [file pone.0221320.s001.pdf]

Table S1. Mean values of individual participant in full visual condition in younger, middle-aged and older groups

Group	Participant	TMT (ms)	MV (cm/ms)	TMV (ms)	%TMV	DT (ms)	MA (cm)	TMA (ms)	%TMA	Tmax (ms)	rmax
**Younger group**	Y01	375.33	145.16	154.67	40.74	220.67	8.40	250.00	66.66	96.00	0.88
	Y02	374.67	185.86	106.67	28.74	268.00	8.98	215.33	57.74	104.67	0.88
	Y03	404.67	136.38	142.00	35.31	262.67	10.39	240.67	59.55	106.00	0.83
	Y04	388.00	127.62	155.33	39.82	232.67	9.42	254.67	65.61	113.33	0.90
	Y05	402.00	147.77	174.00	43.29	228.00	9.19	276.00	69.09	80.00	0.94
	Y06	460.00	173.10	128.67	28.75	331.33	11.50	236.67	53.01	129.33	0.92
	Y07	425.33	141.37	118.67	27.84	306.67	9.57	286.00	67.13	136.67	0.72
	Y08	426.67	151.45	146.67	34.42	280.00	10.71	284.67	66.71	112.67	0.93
	Y09	550.67	114.42	211.33	38.48	339.33	8.12	329.33	59.68	131.33	0.94
	Y10	354.67	190.53	116.67	33.10	238.00	12.84	206.00	58.27	77.33	0.94
	Y11	390.00	153.53	208.67	53.53	181.33	10.23	279.33	71.62	44.00	0.92
	Y12	448.00	121.53	143.33	32.53	304.67	10.48	267.33	60.60	98.00	0.88
	**Mean**	**416.66**	**149.05**	**150.55**	**36.37**	**266.11**	**9.98**	**260.50**	**62.97**	**102.44**	**0.89**
	**SD**	**52.39**	**23.99**	**33.53**	**7.36**	**48.21**	**1.33**	**34.01**	**5.56**	**26.25**	**0.06**

Group	Participant	TMT (ms)	MV (cm/ms)	TMV (ms)	%TMV	DT (ms)	MA (cm)	TMA (ms)	%TMA	Tmax (ms)	rmax
**Middle-aged group**	M01	565.33	94.73	242.00	42.77	323.33	7.38	426.00	75.24	175.33	0.87
	M02	556.67	97.70	136.00	24.67	420.67	6.90	349.33	62.97	186.67	0.70
	M03	543.33	104.75	160.00	29.46	383.33	9.01	390.00	71.76	156.67	0.85
	M04	607.33	80.54	240.00	39.49	367.33	7.63	430.00	71.15	160.00	0.87
	M05	466.67	108.19	231.33	49.81	235.33	11.90	350.00	75.09	126.00	0.80
	M06	492.00	106.32	191.33	38.72	300.67	7.39	344.00	69.73	131.33	0.92
	M07	680.67	107.47	276.67	40.59	404.00	8.46	450.67	66.23	186.00	0.94
	M08	458.67	120.90	221.33	48.30	237.33	10.04	359.33	78.27	150.00	0.78
	M09	566.00	97.68	246.67	43.54	319.33	9.37	396.67	70.23	127.33	0.84
	M10	650.67	91.05	295.33	45.34	355.33	8.14	476.67	73.29	164.00	0.90
	M11	427.33	124.80	201.33	47.02	226.00	8.26	326.00	76.38	129.33	0.84
	M12	685.33	90.99	308.00	45.06	377.33	11.16	522.00	76.23	184.00	0.89
	**Mean**	**558.33**	**102.09**	**229.16**	**41.22**	**329.16**	**8.80**	**401.72**	**72.21**	**156.38**	**0.85**
	**SD**	**86.47**	**12.65**	**51.49**	**7.49**	**67.54**	**1.56**	**60.53**	**4.48**	**23.65**	**0.06**

Group	Participant	TMT (ms)	MV (cm/ms)	TMV (ms)	%TMV	DT (ms)	MA (cm)	TMA (ms)	%TMA	Tmax (ms)	rmax
**Older group**	O01	630.67	96.35	248.00	39.31	382.67	4.91	433.33	68.78	180.00	0.76
	O02	654.67	87.34	265.33	40.53	389.33	6.24	495.33	75.84	194.67	0.92
	O03	478.67	101.25	174.00	36.34	304.67	9.06	336.67	70.54	123.33	0.86
	O04	630.00	101.46	283.33	45.01	346.67	7.66	476.00	75.67	179.33	0.85
	O05	632.67	93.35	252.00	40.35	380.67	10.75	266.67	41.93	58.00	0.86
	O06	483.33	120.33	132.00	27.86	351.33	8.89	322.00	67.29	195.33	0.80
	O07	818.00	90.95	363.33	44.44	454.67	8.23	618.00	75.56	254.67	0.86
	O08	707.33	105.55	270.67	38.64	436.67	7.89	471.33	67.41	180.67	0.92
	O09	584.67	88.93	236.00	40.31	348.67	7.04	384.67	65.70	175.33	0.84
	O10	752.00	94.04	219.33	30.27	532.67	8.08	532.00	71.34	262.00	0.91
	O11	505.33	121.03	194.67	38.62	310.67	10.16	337.33	66.94	142.67	0.93
	O12	453.33	109.73	231.33	50.99	222.00	9.59	324.67	71.56	105.33	0.75
	**Mean**	**610.88**	**100.85**	**239.16**	**39.38**	**371.72**	**8.20**	**416.50**	**68.21**	**170.94**	**0.85**
	**SD**	**115.07**	**11.41**	**58.35**	**6.18**	**79.61**	**1.64**	**104.64**	**9.01**	**57.88**	**0.06**
