# Supplementary material for: Age-related changes in reach-to-grasp movements with partial visual occlusion
Source: PLoS One. 2019 Aug 28;14(8):e0221320. doi: 10.1371/journal.pone.0221320 (PMC6713340; doi:10.1371/journal.pone.0221320)
Supplement: S2 Table — Mean values of individual participant in visual occlusion condition for younger, middle-aged and older groups. (PDF) [file pone.0221320.s002.pdf]

Table S2. Mean values of individual participant in visual occlusion condition in younger, middle-aged and older groups

	Participant	TMT (ms)	MV (cm/ms)	TMV (ms)	%TMV	DT (ms)	MA (cm)	TMA (ms)	%TMA	Tmax (ms)	rmax
**Younger group**	Y01	405.33	143.61	152.67	37.86	252.67	9.79	251.33	62.52	111.33	0.88
	Y02	379.33	161.02	102.00	26.82	277.33	10.76	236.67	62.19	96.00	0.86
	Y03	454.67	119.82	170.00	37.22	284.67	10.77	280.00	61.60	128.00	0.83
	Y04	390.00	116.75	153.33	39.37	236.67	10.82	276.67	70.82	98.00	0.85
	Y05	362.67	137.49	159.33	43.94	203.33	10.07	260.00	71.72	72.67	0.92
	Y06	366.00	173.14	105.33	29.05	260.67	12.20	224.67	61.59	89.33	0.89
	Y07	497.33	108.42	168.00	33.88	329.33	9.25	338.00	67.94	176.00	0.70
	Y08	470.67	117.33	183.33	39.93	287.33	8.61	320.67	68.26	136.67	0.81
	Y09	507.33	110.02	198.00	39.25	309.33	8.17	352.00	69.43	179.33	0.85
	Y10	433.33	134.14	96.00	22.14	337.33	12.33	294.00	67.87	100.67	0.93
	Y11	354.67	159.73	216.00	60.84	138.67	10.76	255.33	71.87	30.00	0.87
	Y12	425.33	120.06	175.33	41.19	250.00	10.94	282.67	66.45	90.67	0.83
	**Mean**	**420.55**	**133.45**	**156.61**	**37.62**	**263.94**	**10.37**	**281.00**	**66.85**	**109.05**	**0.85**
	**SD**	**52.93**	**21.79**	**38.05**	**9.75**	**55.03**	**1.27**	**39.51**	**3.94**	**41.79**	**0.05**

	Participant	TMT (ms)	MV (cm/ms)	TMV (ms)	%TMV	DT (ms)	MA (cm)	TMA (ms)	%TMA	Tmax (ms)	rmax
**Middle-aged group**	M01	640.67	89.62	314.67	49.09	326.00	8.10	468.00	73.06	158.67	0.83
	M02	552.00	96.29	208.00	37.47	344.00	7.25	378.67	68.71	179.33	0.67
	M03	607.33	98.26	202.67	33.34	404.67	7.70	423.33	69.60	199.33	0.91
	M04	625.33	82.16	265.33	42.54	360.00	8.11	438.67	70.16	157.33	0.92
	M05	532.67	95.81	268.00	50.47	264.67	11.85	394.67	74.20	158.67	0.81
	M06	577.33	89.54	259.33	44.95	318.00	7.00	400.67	69.28	161.33	0.89
	M07	813.33	86.66	345.33	42.62	468.00	8.23	538.67	66.45	241.33	0.83
	M08	566.67	99.77	279.33	50.06	287.33	10.20	420.00	74.55	142.00	0.86
	M09	635.33	88.53	311.33	49.38	324.00	8.04	480.67	75.84	151.33	0.82
	M10	735.33	83.85	402.67	54.72	332.67	7.78	574.00	78.13	144.67	0.75
	M11	687.33	89.23	295.33	43.05	392.00	10.52	517.33	75.33	168.67	0.93
	M12	688.67	88.68	296.00	43.00	392.67	10.49	513.33	74.54	169.33	0.93
	**Mean**	**638.50**	**90.70**	**287.33**	**45.05**	**351.16**	**8.77**	**462.33**	**72.48**	**169.33**	**0.84**
	**SD**	**81.79**	**5.61**	**55.01**	**6.01**	**55.92**	**1.56**	**62.91**	**3.53**	**27.46**	**0.07**

	Participant	TMT (ms)	MV (cm/ms)	TMV (ms)	%TMV	DT (ms)	MA (cm)	TMA (ms)	%TMA	Tmax (ms)	rmax
**Older group**	O01	655.33	80.08	302.00	46.05	353.33	5.01	460.67	69.99	142.67	0.76
	O02	652.67	75.89	264.67	40.52	388.00	6.13	508.00	77.81	189.33	0.89
	O03	494.67	98.32	185.33	37.17	309.33	10.02	348.00	70.31	143.33	0.84
	O04	795.33	82.20	336.00	42.34	459.33	7.74	568.67	71.55	228.67	0.91
	O05	756.00	83.86	331.33	44.35	424.67	7.20	512.00	68.06	214.00	0.77
	O06	532.67	100.70	208.67	39.70	324.00	10.07	384.00	72.27	220.67	0.65
	O07	900.67	72.17	439.33	49.57	461.33	8.65	676.00	75.45	242.00	0.75
	O08	633.33	94.04	228.67	36.19	404.67	7.18	406.67	64.62	154.67	0.90
	O09	626.00	83.22	293.33	47.03	332.67	7.77	424.00	67.91	155.33	0.84
	O10	694.67	109.77	154.00	22.45	540.67	8.10	446.67	64.85	234.67	0.89
	O11	544.00	115.26	216.00	39.65	328.00	10.12	369.33	67.93	142.67	0.91
	O12	568.67	97.00	282.00	49.61	286.67	9.26	415.33	73.13	152.67	0.86
	**Mean**	**654.50**	**91.04**	**270.11**	**41.21**	**384.38**	**8.10**	**459.94**	**70.32**	**185.05**	**0.83**
	**SD**	**118.15**	**13.56**	**78.35**	**7.43**	**76.07**	**1.61**	**93.57**	**3.98**	**40.36**	**0.08**

Total movement time (TMT), maximum transport velocity (MV), absolute time of maximum transport velocity (TMV), relative time of maximum transport velocity (% TMV), deceleration time (DT), maximum aperture (MA), absolute time of maximum aperture (TMA), relative time of maximum aperture (% TMA), maximum time lag (Tmax), maximum correlation coefficient (rmax)
