# Supplementary material for: Age-related changes in reach-to-grasp movements with partial visual occlusion
Source: PLoS One. 2019 Aug 28;14(8):e0221320. doi: 10.1371/journal.pone.0221320 (PMC6713340; doi:10.1371/journal.pone.0221320)
Supplement: S3 Table — Raw data of individual participant in full visual condition for younger, middle-aged and older groups. (PDF) [file pone.0221320.s003.pdf]

Table S3. Raw data of individual participant in full visual condition in younger, middle-aged and older groups

Participant	Trial no.	TMT (ms)	MV (cm/ms)	TMV (ms)	%TMV	DT (ms)	MA (cm)	TMA (ms)	%TMA
Y01	3	460.00	130.11	210.00	45.65	250.00	7.88	300.00	65.22
full vision	4	530.00	117.32	280.00	52.83	250.00	7.57	360.00	67.93
	5	390.00	143.98	170.00	43.59	220.00	8.36	220.00	56.41
(Younger)	18	360.00	150.98	150.00	41.67	210.00	10.15	240.00	66.67
	7	370.00	131.02	170.00	45.95	200.00	7.18	260.00	70.27
	8	340.00	138.61	120.00	35.29	220.00	8.39	260.00	76.47
	9	350.00	152.23	130.00	37.14	220.00	7.64	220.00	62.86
	10	340.00	167.14	150.00	44.12	190.00	9.74	230.00	67.65
	11	330.00	141.70	150.00	45.46	180.00	8.42	220.00	66.67
	12	340.00	167.57	120.00	35.29	220.00	7.48	210.00	61.77
	13	360.00	146.33	130.00	36.11	230.00	8.19	270.00	75.00
	14	360.00	151.84	110.00	30.56	250.00	8.79	220.00	61.11
	15	330.00	163.42	130.00	39.39	200.00	8.40	220.00	66.67
	16	390.00	134.56	140.00	35.90	250.00	9.38	250.00	64.10
	17	380.00	140.56	160.00	42.11	220.00	8.51	270.00	71.05
	max	530.00	167.57	280.00	52.83	250.00	10.15	360.00	76.47
	mean	375.33	145.16	154.67	40.74	220.67	8.40	250.00	66.66
	SD	53.97	14.24	42.91	5.76	22.51	0.84	39.82	5.23

Participant	Trial no.	TMT (ms)	MV (cm/ms)	TMV (ms)	%TMV	DT (ms)	MA (cm)	TMA (ms)	%TMA
Y02	3	510.00	128.39	110.00	21.57	400.00	8.54	200.00	39.22
full vision	4	460.00	114.76	130.00	28.26	330.00	8.04	340.00	73.91
	18	350.00	235.08	100.00	28.57	250.00	9.65	170.00	48.57
	6	350.00	186.91	110.00	31.43	240.00	7.93	220.00	62.86
	7	410.00	156.44	130.00	31.71	280.00	8.35	230.00	56.10
	8	370.00	204.31	100.00	27.03	270.00	7.53	250.00	67.57
	9	350.00	206.46	110.00	31.43	240.00	9.42	200.00	57.14
	10	340.00	196.48	90.00	26.47	250.00	9.47	200.00	58.82
	11	350.00	199.99	100.00	28.57	250.00	10.75	200.00	57.14
	12	360.00	205.26	100.00	27.78	260.00	8.38	200.00	55.56
	13	360.00	175.33	110.00	30.56	250.00	9.43	180.00	50.00
	14	310.00	195.94	100.00	32.26	210.00	9.18	170.00	54.84
	15	410.00	181.39	110.00	26.83	300.00	9.83	230.00	56.10
	16	310.00	206.89	100.00	32.26	210.00	9.45	210.00	67.74
	17	380.00	194.20	100.00	26.32	280.00	8.70	230.00	60.53
	max	510.00	235.08	130.00	32.26	400.00	10.75	340.00	73.91
	mean	374.67	185.86	106.67	28.74	268.00	8.98	215.33	57.74
	SD	53.83	31.45	11.13	2.95	47.99	0.86	41.38	8.45

Participant	Trial no.	TMT (ms)	MV (cm/ms)	TMV (ms)	%TMV	DT (ms)	MA (cm)	TMA (ms)	%TMA
Y03	3	470.00	141.83	150.00	31.92	320.00	9.46	290.00	61.70
full vision	4	400.00	147.23	130.00	32.50	270.00	9.63	270.00	67.50
	5	440.00	146.22	110.00	25.00	330.00	9.13	220.00	50.00
	6	390.00	161.22	100.00	25.64	290.00	11.38	230.00	58.97
	7	420.00	120.98	90.00	21.43	330.00	10.95	260.00	61.91
	8	400.00	132.52	200.00	50.00	200.00	11.15	220.00	55.00
	9	470.00	101.90	160.00	34.04	310.00	9.35	280.00	59.57
	10	410.00	154.91	120.00	29.27	290.00	12.74	230.00	56.10
	11	370.00	128.87	100.00	27.03	270.00	10.60	230.00	62.16
	12	430.00	131.83	190.00	44.19	240.00	8.77	250.00	58.14
	13	390.00	118.44	160.00	41.03	230.00	9.12	240.00	61.54
	14	390.00	127.80	190.00	48.72	200.00	10.22	240.00	61.54
	15	350.00	137.22	170.00	48.57	180.00	11.74	220.00	62.86
	16	360.00	137.91	130.00	36.11	230.00	11.45	210.00	58.33
	17	380.00	156.83	130.00	34.21	250.00	10.19	220.00	57.90
	max	470.00	161.22	200.00	50.00	330.00	12.74	290.00	67.50
	mean	404.67	136.38	142.00	35.31	262.67	10.39	240.67	59.55
	SD	36.03	15.90	35.50	9.27	49.06	1.16	24.34	4.05

Participant	Trial no.	TMT (ms)	MV (cm/ms)	TMV (ms)	%TMV	DT (ms)	MA (cm)	TMA (ms)	%TMA
Y04	3	410.00	107.76	240.00	58.54	170.00	9.03	290.00	70.73
full vision	4	380.00	124.74	130.00	34.21	250.00	9.89	240.00	63.16
	5	370.00	126.95	140.00	37.84	230.00	9.46	270.00	72.97
	6	380.00	135.20	140.00	36.84	240.00	9.46	270.00	71.05
	7	380.00	131.94	150.00	39.47	230.00	9.15	260.00	68.42
	8	380.00	130.54	160.00	42.11	220.00	9.53	230.00	60.53
	9	380.00	115.66	200.00	52.63	180.00	9.43	270.00	71.05
	10	360.00	123.29	120.00	33.33	240.00	9.25	230.00	63.89
	11	410.00	114.71	150.00	36.59	260.00	9.66	270.00	65.85
	12	380.00	143.18	130.00	34.21	250.00	9.02	240.00	63.16
	13	430.00	109.37	220.00	51.16	210.00	9.41	290.00	67.44
	14	440.00	125.30	180.00	40.91	260.00	10.12	280.00	63.64
	15	390.00	144.65	120.00	30.77	270.00	8.60	220.00	56.41
	16	340.00	149.90	120.00	35.29	220.00	9.79	210.00	61.77
	17	390.00	131.10	130.00	33.33	260.00	9.49	250.00	64.10
	max	440.00	149.90	240.00	58.54	270.00	10.12	290.00	72.97
	mean	388.00	127.62	155.33	39.82	232.67	9.42	254.67	65.61
	SD	25.69	12.52	37.96	8.12	29.15	0.38	25.32	4.61

Participant	Trial no.	TMT (ms)	MV (cm/ms)	TMV (ms)	%TMV	DT (ms)	MA (cm)	TMA (ms)	%TMA
Y05	3	470.00	129.83	260.00	55.32	210.00	7.34	390.00	82.98
full vision	4	420.00	126.36	200.00	47.62	220.00	8.89	300.00	71.43
	5	410.00	139.49	200.00	48.78	210.00	9.37	280.00	68.29
	6	420.00	147.23	190.00	45.24	230.00	9.59	290.00	69.05
	7	400.00	161.27	180.00	45.00	220.00	9.62	290.00	72.50
	8	410.00	146.88	150.00	36.59	260.00	9.43	290.00	70.73
	9	530.00	125.18	180.00	33.96	350.00	7.98	230.00	43.40
	10	410.00	155.60	190.00	46.34	220.00	9.76	290.00	70.73
	11	380.00	147.24	150.00	39.47	230.00	9.57	270.00	71.05
	12	400.00	136.62	150.00	37.50	250.00	9.16	280.00	70.00
	13	350.00	164.40	160.00	45.71	190.00	9.66	240.00	68.57
	14	340.00	182.06	130.00	38.24	210.00	9.92	240.00	70.59
	15	350.00	145.41	170.00	48.57	180.00	9.32	250.00	71.43
	16	390.00	148.66	160.00	41.03	230.00	9.46	250.00	64.10
	17	350.00	160.28	140.00	40.00	210.00	8.78	250.00	71.43
	max	530.00	182.06	260.00	55.32	350.00	9.92	390.00	82.98
	mean	402.00	147.77	174.00	43.29	228.00	9.19	276.00	69.09
	SD	49.60	15.45	32.25	5.76	39.32	0.70	38.88	8.10

Participant	Trial no.	TMT (ms)	MV (cm/ms)	TMV (ms)	%TMV	DT (ms)	MA (cm)	TMA (ms)	%TMA
Y06	3	440.00	178.49	150.00	34.09	290.00	10.26	270.00	61.36
full vision	4	440.00	144.80	110.00	25.00	330.00	11.14	260.00	59.09
	5	420.00	159.50	170.00	40.48	250.00	12.34	240.00	57.14
	6	430.00	153.54	110.00	25.58	320.00	11.47	280.00	65.12
	7	550.00	167.65	150.00	27.27	400.00	12.17	220.00	40.00
	8	390.00	174.16	100.00	25.64	290.00	12.00	250.00	64.10
	9	380.00	164.18	100.00	26.32	280.00	11.40	260.00	68.42
	10	600.00	150.25	90.00	15.00	510.00	10.48	240.00	40.00
	11	560.00	173.77	150.00	26.79	410.00	11.87	220.00	39.29
	12	550.00	158.79	90.00	16.36	460.00	11.33	240.00	43.64
	13	410.00	201.33	160.00	39.02	250.00	10.95	220.00	53.66
	14	500.00	192.03	140.00	28.00	360.00	12.40	200.00	40.00
	15	510.00	189.50	150.00	29.41	360.00	11.98	210.00	41.18
	16	360.00	208.94	170.00	47.22	190.00	11.33	240.00	66.67
	17	360.00	179.61	90.00	25.00	270.00	11.42	200.00	55.56
	max	600.00	208.94	170.00	47.22	510.00	12.40	280.00	68.42
	mean	460.00	173.10	128.67	28.75	331.33	11.50	236.67	53.01
	SD	78.83	18.87	30.67	8.52	86.26	0.64	24.69	11.18

Participant	Trial no.	TMT (ms)	MV (cm/ms)	TMV (ms)	%TMV	DT (ms)	MA (cm)	TMA (ms)	%TMA
Y07	3	440.00	109.10	200.00	45.46	240.00	10.15	290.00	65.91
full vision	4	440.00	110.04	110.00	25.00	330.00	9.33	320.00	72.73
	5	470.00	119.21	120.00	25.53	160.00	8.64	330.00	70.21
	6	440.00	123.15	120.00	27.27	320.00	8.97	250.00	56.82
	7	470.00	117.60	130.00	27.66	170.00	9.17	360.00	76.60
	8	430.00	153.38	120.00	27.91	150.00	11.06	270.00	62.79
	9	440.00	139.95	110.00	25.00	330.00	8.95	310.00	70.46
	10	400.00	150.76	100.00	25.00	300.00	10.30	240.00	60.00
	11	390.00	145.41	100.00	25.64	290.00	9.96	250.00	64.10
	12	410.00	140.49	110.00	26.83	300.00	9.81	250.00	60.98
	13	430.00	143.13	130.00	30.23	150.00	9.16	320.00	74.42
	14	420.00	167.90	110.00	26.19	150.00	9.67	260.00	61.91
	15	410.00	177.17	120.00	29.27	150.00	9.82	290.00	70.73
	16	390.00	156.70	100.00	25.64	290.00	8.01	270.00	69.23
	17	400.00	166.53	100.00	25.00	150.00	10.59	280.00	70.00
	max	470.00	177.17	200.00	45.46	330.00	11.06	360.00	76.60
	mean	425.33	141.37	118.67	27.84	232.00	9.57	286.00	67.13
	SD	25.60	21.58	24.75	5.14	78.21	0.79	35.42	5.81

Participant	Trial no.	TMT (ms)	MV (cm/ms)	TMV (ms)	%TMV	DT (ms)	MA (cm)	TMA (ms)	%TMA
Y08	3	410.00	144.47	130.00	31.71	280.00	10.30	290.00	70.73
full vision	4	420.00	148.67	110.00	26.19	310.00	10.39	280.00	66.67
	5	410.00	162.32	130.00	31.71	280.00	10.64	290.00	70.73
	6	470.00	147.53	120.00	25.53	350.00	10.15	280.00	59.57
	7	440.00	152.45	210.00	47.73	230.00	9.97	310.00	70.46
	8	420.00	154.33	140.00	33.33	280.00	10.54	280.00	66.67
	9	450.00	166.64	180.00	40.00	270.00	11.22	320.00	71.11
	10	440.00	151.22	120.00	27.27	320.00	10.37	260.00	59.09
	11	410.00	126.89	190.00	46.34	220.00	10.00	240.00	58.54
	12	400.00	151.97	120.00	30.00	280.00	10.53	270.00	67.50
	13	440.00	152.00	190.00	43.18	250.00	10.95	320.00	72.73
	14	430.00	151.24	120.00	27.91	310.00	11.59	290.00	67.44
	15	430.00	148.56	140.00	32.56	290.00	10.96	300.00	69.77
	16	440.00	165.73	140.00	31.82	300.00	11.91	300.00	68.18
	17	390.00	147.73	160.00	41.03	230.00	11.14	240.00	61.54
	max	470.00	166.64	210.00	47.73	350.00	11.91	320.00	72.73
	mean	426.67	151.45	146.67	34.42	280.00	10.71	284.67	66.71
	SD	20.93	9.49	31.55	7.35	36.25	0.57	24.75	4.76

Participant	Trial no.	TMT (ms)	MV (cm/ms)	TMV (ms)	%TMV	DT (ms)	MA (cm)	TMA (ms)	%TMA
Y09	3	650.00	106.51	230.00	35.39	420.00	7.24	460.00	70.77
full vision	4	590.00	114.79	230.00	38.98	360.00	7.90	340.00	57.63
	5	550.00	119.73	200.00	36.36	350.00	7.98	250.00	45.46
	6	580.00	108.23	170.00	29.31	410.00	8.44	350.00	60.35
	7	530.00	125.14	140.00	26.42	390.00	8.25	250.00	47.17
	8	510.00	124.50	230.00	45.10	280.00	9.01	330.00	64.71
	9	520.00	117.82	220.00	42.31	300.00	8.83	340.00	65.39
	10	570.00	121.30	230.00	40.35	340.00	8.91	330.00	57.90
	11	530.00	111.11	230.00	43.40	300.00	8.09	310.00	58.49
	12	520.00	112.94	190.00	36.54	330.00	7.19	320.00	61.54
	13	530.00	113.35	210.00	39.62	320.00	8.19	290.00	54.72
	14	540.00	105.18	230.00	42.59	310.00	7.84	350.00	64.82
	15	520.00	127.31	210.00	40.39	310.00	8.35	280.00	53.85
	16	540.00	104.78	230.00	42.59	310.00	8.33	380.00	70.37
	17	580.00	103.55	220.00	37.93	360.00	7.22	360.00	62.07
	max	650.00	127.31	230.00	45.10	420.00	9.01	460.00	70.77
	mean	550.67	114.42	211.33	38.48	339.33	8.12	329.33	59.68
	SD	37.31	7.94	26.69	5.16	42.00	0.58	52.57	7.36

Participant	Trial no.	TMT (ms)	MV (cm/ms)	TMV (ms)	%TMV	DT (ms)	MA (cm)	TMA (ms)	%TMA
Y10	3	370.00	196.25	110.00	29.73	260.00	11.95	260.00	70.27
full vision	4	380.00	196.22	140.00	36.84	240.00	12.43	200.00	52.63
	5	350.00	168.75	90.00	25.71	260.00	12.93	210.00	60.00
	6	380.00	199.49	90.00	23.68	290.00	12.90	210.00	55.26
	7	360.00	198.87	150.00	41.67	210.00	12.34	210.00	58.33
	8	330.00	199.31	120.00	36.36	210.00	12.86	190.00	57.58
	9	360.00	212.29	70.00	19.44	290.00	12.55	190.00	52.78
	10	330.00	157.04	140.00	42.42	190.00	12.09	220.00	66.67
	11	330.00	208.53	140.00	42.42	190.00	12.14	200.00	60.61
	12	350.00	211.28	130.00	37.14	220.00	13.33	190.00	54.29
	13	370.00	181.93	80.00	21.62	290.00	13.17	190.00	51.35
	14	320.00	198.43	140.00	43.75	180.00	13.18	190.00	59.38
	15	340.00	168.09	80.00	23.53	260.00	13.35	240.00	70.59
	16	360.00	181.58	140.00	38.89	220.00	13.26	200.00	55.56
	17	390.00	179.84	130.00	33.33	260.00	14.15	190.00	48.72
	max	390.00	212.29	150.00	43.75	290.00	14.15	260.00	70.59
	mean	354.67	190.53	116.67	33.10	238.00	12.84	206.00	58.27
	SD	21.34	16.80	27.43	8.45	38.02	0.60	20.63	6.60

Participant	Trial no.	TMT (ms)	MV (cm/ms)	TMV (ms)	%TMV	DT (ms)	MA (cm)	TMA (ms)	%TMA
Y11	3	410.00	130.55	200.00	48.78	210.00	10.55	300.00	73.17
full vision	4	380.00	138.85	210.00	55.26	170.00	9.45	240.00	63.16
	5	410.00	143.75	220.00	53.66	190.00	10.63	290.00	70.73
	6	380.00	141.50	210.00	55.26	170.00	10.36	280.00	73.68
	7	540.00	110.01	300.00	55.56	240.00	9.23	390.00	72.22
	8	370.00	166.39	210.00	56.76	160.00	11.17	260.00	70.27
	9	390.00	161.32	230.00	58.97	160.00	10.21	290.00	74.36
	10	350.00	164.76	190.00	54.29	160.00	10.38	260.00	74.29
	11	340.00	162.07	180.00	52.94	160.00	10.73	250.00	73.53
	12	390.00	155.06	230.00	58.97	160.00	9.92	290.00	74.36
	13	390.00	151.60	90.00	23.08	300.00	10.24	290.00	74.36
	14	370.00	186.76	200.00	54.05	170.00	10.20	210.00	56.76
	15	360.00	158.94	220.00	61.11	140.00	10.29	280.00	77.78
	16	370.00	153.69	210.00	56.76	160.00	9.80	280.00	75.68
	17	400.00	177.64	230.00	57.50	170.00	10.31	280.00	70.00
	mean	390.00	153.53	208.67	53.53	181.33	10.23	279.33	71.62
	SD	46.14	18.94	42.57	8.92	40.86	0.49	38.82	5.29
	max	540.00	186.76	300.00	61.11	300.00	11.17	390.00	77.78

Participant	Trial no.	TMT (ms)	MV (cm/ms)	TMV (ms)	%TMV	DT (ms)	MA (cm)	TMA (ms)	%TMA
Y12	3	590.00	114.73	120.00	20.34	470.00	9.31	290.00	49.15
full vision	4	390.00	121.86	110.00	28.21	280.00	10.60	230.00	58.97
	5	440.00	105.53	120.00	27.27	320.00	11.39	270.00	61.36
	6	400.00	122.88	190.00	47.50	210.00	10.78	260.00	65.00
	7	420.00	122.76	170.00	40.48	250.00	9.70	290.00	69.05
	8	410.00	120.25	120.00	29.27	290.00	10.75	260.00	63.42
	9	640.00	125.14	140.00	21.88	500.00	10.40	260.00	40.63
	10	390.00	142.05	120.00	30.77	270.00	11.60	240.00	61.54
	11	490.00	101.16	240.00	48.98	250.00	9.46	310.00	63.27
	12	440.00	139.63	130.00	29.55	310.00	11.02	260.00	59.09
	13	400.00	116.82	140.00	35.00	260.00	11.20	250.00	62.50
	14	440.00	114.79	150.00	34.09	290.00	10.46	280.00	63.64
	15	410.00	120.70	130.00	31.71	280.00	10.40	260.00	63.42
	16	420.00	134.35	140.00	33.33	280.00	10.25	270.00	64.29
	17	440.00	120.36	130.00	29.55	310.00	9.83	280.00	63.64
	mean	448.00	121.53	143.33	32.53	304.67	10.48	267.33	60.60
	SD	73.11	11.08	33.95	8.05	78.46	0.69	20.52	7.00
	max	640.00	142.05	240.00	48.98	500.00	11.60	310.00	69.05

Participant	Trial no.	TMT (ms)	MV (cm/ms)	TMV (ms)	%TMV	DT (ms)	MA (cm)	TMA (ms)	%TMA
M01	3	500.00	95.80	230.00	46.00	270.00	7.73	350.00	70.00
full vision	4	550.00	93.02	130.00	23.64	420.00	6.62	420.00	76.36
	5	650.00	90.82	250.00	38.46	400.00	6.71	510.00	78.46
(Middle-aged)	6	470.00	107.70	200.00	42.55	270.00	8.42	350.00	74.47
	7	600.00	98.68	250.00	41.67	350.00	7.65	420.00	70.00
	8	660.00	90.78	310.00	46.97	350.00	6.25	480.00	72.73
	9	550.00	93.03	270.00	49.09	280.00	8.01	410.00	74.55
	10	620.00	89.66	260.00	41.94	360.00	6.90	470.00	75.81
	11	580.00	91.23	280.00	48.28	300.00	7.91	460.00	79.31
	12	550.00	97.11	240.00	43.64	310.00	7.29	400.00	72.73
	13	550.00	91.31	220.00	40.00	330.00	7.61	400.00	72.73
	14	680.00	84.17	320.00	47.06	360.00	6.75	540.00	79.41
	15	470.00	96.19	240.00	51.06	230.00	8.25	330.00	70.21
	16	510.00	103.44	140.00	27.45	370.00	7.05	410.00	80.39
	17	540.00	97.99	290.00	53.70	250.00	7.51	440.00	81.48
	max	680.00	107.70	320.00	53.70	420.00	8.42	540.00	81.48
	mean	565.33	94.73	242.00	42.77	323.33	7.38	426.00	75.24
	SD	65.89	5.84	54.14	8.17	56.02	0.65	59.14	3.89

Participant	Trial no.	TMT (ms)	MV (cm/ms)	TMV (ms)	%TMV	DT (ms)	MA (cm)	TMA (ms)	%TMA
M02	3	610.00	92.97	130.00	21.31	480.00	7.55	370.00	60.66
full vision	4	560.00	109.35	100.00	17.86	460.00	8.16	310.00	55.36
	5	620.00	82.20	110.00	17.74	510.00	6.24	400.00	64.52
	6	610.00	89.67	140.00	22.95	470.00	6.74	360.00	59.02
	7	510.00	96.51	100.00	19.61	410.00	7.06	320.00	62.75
	8	610.00	90.37	110.00	18.03	500.00	6.09	410.00	67.21
	9	540.00	94.18	210.00	38.89	330.00	6.62	320.00	59.26
	18	640.00	93.84	110.00	17.19	530.00	6.88	320.00	50.00
	11	530.00	93.97	110.00	20.76	420.00	6.77	340.00	64.15
	12	470.00	100.07	100.00	21.28	370.00	7.33	320.00	68.09
	13	560.00	83.89	100.00	17.86	460.00	5.92	330.00	58.93
	14	540.00	107.79	120.00	22.22	420.00	6.99	370.00	68.52
	15	580.00	95.70	250.00	43.10	330.00	6.85	430.00	74.14
	16	470.00	117.70	100.00	21.28	370.00	6.89	310.00	65.96
	17	500.00	117.26	250.00	50.00	250.00	7.47	330.00	66.00
	max	640.00	117.70	250.00	50.00	530.00	8.16	430.00	74.14
	mean	556.67	97.70	136.00	24.67	420.67	6.90	349.33	62.97
	SD	54.60	10.84	54.09	10.38	78.42	0.58	38.82	5.97

Participant	Trial no.	TMT (ms)	MV (cm/ms)	TMV (ms)	%TMV	DT (ms)	MA (cm)	TMA (ms)	%TMA
M03	3	550.00	91.87	120.00	21.82	430.00	10.90	390.00	70.91
full vision	4	560.00	106.09	200.00	35.71	360.00	10.40	370.00	66.07
	5	530.00	103.49	140.00	26.42	390.00	8.77	370.00	69.81
	6	630.00	96.66	160.00	25.40	470.00	8.47	470.00	74.60
	7	560.00	111.63	140.00	25.00	420.00	8.58	450.00	80.36
	8	450.00	134.35	110.00	24.44	340.00	9.91	320.00	71.11
	18	590.00	104.62	170.00	28.81	420.00	7.44	420.00	71.19
	10	530.00	109.31	140.00	26.42	390.00	8.03	410.00	77.36
	11	510.00	105.23	160.00	31.37	350.00	8.82	360.00	70.59
	12	510.00	96.63	180.00	35.29	330.00	9.25	370.00	72.55
	13	540.00	105.51	120.00	22.22	420.00	8.80	360.00	66.67
	14	550.00	92.84	160.00	29.09	390.00	9.41	380.00	69.09
	15	530.00	97.41	200.00	37.74	330.00	8.61	410.00	77.36
	16	570.00	115.92	190.00	33.33	380.00	8.96	390.00	68.42
	17	540.00	99.73	210.00	38.89	330.00	8.84	380.00	70.37
	max	630.00	134.35	210.00	38.89	470.00	10.90	470.00	80.36
	mean	543.33	104.75	160.00	29.46	383.33	9.01	390.00	71.76
	SD	40.12	10.66	31.62	5.61	43.04	0.88	37.61	4.06

Participant	Trial no.	TMT (ms)	MV (cm/ms)	TMV (ms)	%TMV	DT (ms)	MA (cm)	TMA (ms)	%TMA
M04	3	560.00	85.25	220.00	39.29	340.00	8.09	380.00	67.86
full vision	4	630.00	68.98	160.00	25.40	470.00	7.71	460.00	73.02
	5	590.00	73.40	260.00	44.07	330.00	7.63	460.00	77.97
	6	820.00	73.68	310.00	37.81	510.00	6.81	530.00	64.63
	7	610.00	78.61	270.00	44.26	340.00	7.23	450.00	73.77
	8	620.00	84.10	280.00	45.16	340.00	7.69	440.00	70.97
	9	550.00	81.92	150.00	27.27	400.00	8.29	420.00	76.36
	10	710.00	85.58	240.00	33.80	470.00	6.60	400.00	56.34
	11	610.00	80.74	320.00	52.46	290.00	7.46	440.00	72.13
	12	520.00	79.94	210.00	40.39	310.00	7.75	370.00	71.15
	13	600.00	73.74	260.00	43.33	340.00	7.88	430.00	71.67
	14	540.00	83.62	120.00	22.22	420.00	7.88	390.00	72.22
	15	520.00	87.63	230.00	44.23	290.00	8.85	370.00	71.15
	16	610.00	85.97	260.00	42.62	350.00	7.24	460.00	75.41
	17	620.00	84.99	310.00	50.00	310.00	7.35	450.00	72.58
	max	820.00	87.63	320.00	52.46	510.00	8.85	530.00	77.97
	mean	607.33	80.54	240.00	39.49	367.33	7.63	430.00	71.15
	SD	76.67	5.69	59.88	8.80	69.95	0.56	43.09	5.21

Participant	Trial no.	TMT (ms)	MV (cm/ms)	TMV (ms)	%TMV	DT (ms)	MA (cm)	TMA (ms)	%TMA
M05	3	470.00	107.49	250.00	53.19	220.00	11.65	360.00	76.60
full vision	4	500.00	111.63	260.00	52.00	240.00	10.84	360.00	72.00
	5	520.00	121.16	230.00	44.23	290.00	12.09	390.00	75.00
	6	510.00	94.74	250.00	49.02	260.00	11.37	380.00	74.51
	7	470.00	101.78	240.00	51.06	230.00	11.41	340.00	72.34
	8	440.00	104.51	240.00	54.55	200.00	12.25	340.00	77.27
	9	490.00	102.70	170.00	34.69	320.00	11.80	360.00	73.47
	10	510.00	99.76	260.00	50.98	250.00	11.28	360.00	70.59
	11	400.00	102.08	240.00	60.00	160.00	11.65	320.00	80.00
	12	440.00	100.46	240.00	54.55	200.00	12.20	330.00	75.00
	13	470.00	111.60	230.00	48.94	240.00	12.48	330.00	70.21
	14	470.00	133.59	190.00	40.43	280.00	11.56	410.00	87.23
	15	420.00	110.41	220.00	52.38	200.00	12.58	320.00	76.19
	16	440.00	115.61	220.00	50.00	220.00	12.43	310.00	70.46
	17	450.00	105.26	230.00	51.11	220.00	12.88	340.00	75.56
	max	520.00	133.59	260.00	60.00	320.00	12.88	410.00	87.23
	mean	466.67	108.19	231.33	49.81	235.33	11.90	350.00	75.09
	SD	35.19	9.78	24.46	6.13	40.68	0.57	28.03	4.36

Participant	Trial no.	TMT (ms)	MV (cm/ms)	TMV (ms)	%TMV	DT (ms)	MA (cm)	TMA (ms)	%TMA
M06	3	490.00	96.24	140.00	28.57	350.00	7.87	390.00	79.59
full vision	4	530.00	103.94	220.00	41.51	310.00	7.33	340.00	64.15
	5	530.00	88.04	280.00	52.83	250.00	6.97	420.00	79.25
	6	540.00	109.22	180.00	33.33	360.00	7.45	410.00	75.93
	7	570.00	87.84	250.00	43.86	320.00	7.46	370.00	64.91
	8	490.00	109.98	220.00	44.90	270.00	8.78	350.00	71.43
	9	510.00	98.94	140.00	27.45	370.00	6.23	400.00	78.43
	10	470.00	111.17	220.00	46.81	250.00	7.40	300.00	63.83
	11	500.00	94.63	240.00	48.00	260.00	7.68	360.00	72.00
	12	490.00	118.05	160.00	32.65	330.00	8.31	330.00	67.35
	13	420.00	125.70	110.00	26.19	310.00	7.55	290.00	69.05
	14	500.00	103.89	210.00	42.00	290.00	6.39	330.00	66.00
	15	430.00	127.75	160.00	37.21	270.00	7.67	240.00	55.81
	16	430.00	109.85	190.00	44.19	240.00	7.12	290.00	67.44
	17	480.00	109.51	150.00	31.25	330.00	6.67	340.00	70.83
	max	570.00	127.75	280.00	52.83	370.00	8.78	420.00	79.59
	mean	492.00	106.32	191.33	38.72	300.67	7.39	344.00	69.73
	SD	42.46	12.01	47.94	8.34	42.67	0.67	50.26	6.65

Participant	Trial no.	TMT (ms)	MV (cm/ms)	TMV (ms)	%TMV	DT (ms)	MA (cm)	TMA (ms)	%TMA
M07	18	700.00	100.01	280.00	40.00	420.00	7.53	430.00	61.43
full vision	4	570.00	110.45	240.00	42.11	330.00	8.53	380.00	66.67
	5	650.00	110.48	230.00	35.39	420.00	8.45	420.00	64.62
	6	610.00	112.08	260.00	42.62	350.00	9.13	430.00	70.49
	7	680.00	101.81	260.00	38.24	420.00	8.45	410.00	60.29
	8	690.00	106.54	280.00	40.58	410.00	8.96	460.00	66.67
	9	680.00	115.70	300.00	44.12	380.00	8.62	470.00	69.12
	10	700.00	107.26	310.00	44.29	390.00	8.75	470.00	67.14
	11	760.00	108.72	310.00	40.79	450.00	8.90	520.00	68.42
	12	760.00	109.61	320.00	42.11	440.00	7.57	490.00	64.47
	13	690.00	107.79	270.00	39.13	420.00	8.38	460.00	66.67
	14	710.00	105.31	300.00	42.25	410.00	8.35	500.00	70.42
	15	720.00	110.05	330.00	45.83	390.00	8.96	480.00	66.67
	16	630.00	100.11	240.00	38.10	390.00	7.78	420.00	66.67
	17	660.00	106.20	220.00	33.33	440.00	8.50	420.00	63.64
	max	760.00	115.70	330.00	45.83	450.00	9.13	520.00	70.49
	mean	680.67	107.47	276.67	40.59	404.00	8.46	450.67	66.23
	SD	51.47	4.38	34.57	3.37	33.12	0.49	38.63	2.94

Participant	Trial no.	TMT (ms)	MV (cm/ms)	TMV (ms)	%TMV	DT (ms)	MA (cm)	TMA (ms)	%TMA
M08	3	470.00	116.05	210.00	44.68	260.00	10.39	360.00	76.60
full vision	4	420.00	123.64	160.00	38.10	260.00	10.20	320.00	76.19
	5	450.00	114.50	260.00	57.78	190.00	10.27	360.00	80.00
	6	470.00	129.72	180.00	38.30	290.00	9.82	370.00	78.72
	7	460.00	106.32	160.00	34.78	300.00	9.84	340.00	73.91
	8	500.00	112.36	270.00	54.00	230.00	9.57	410.00	82.00
	9	430.00	151.17	180.00	41.86	250.00	10.05	330.00	76.74
	10	420.00	125.92	230.00	54.76	190.00	9.25	320.00	76.19
	11	500.00	100.00	280.00	56.00	220.00	9.99	400.00	80.00
	12	440.00	118.11	190.00	43.18	250.00	9.54	340.00	77.27
	13	450.00	119.74	250.00	55.56	200.00	10.26	360.00	80.00
	14	480.00	115.73	200.00	41.67	280.00	9.96	380.00	79.17
	15	430.00	129.22	260.00	60.47	170.00	10.20	340.00	79.07
	16	510.00	119.58	210.00	41.18	300.00	10.80	410.00	80.39
	17	450.00	131.41	280.00	62.22	170.00	10.50	350.00	77.78
	max	510.00	151.17	280.00	62.22	300.00	10.80	410.00	82.00
	mean	458.67	120.90	221.33	48.30	237.33	10.04	359.33	78.27
	SD	29.24	12.04	42.91	9.18	45.59	0.40	29.87	2.12

Participant	Trial no.	TMT (ms)	MV (cm/ms)	TMV (ms)	%TMV	DT (ms)	MA (cm)	TMA (ms)	%TMA
M09	3	630.00	91.05	260.00	41.27	370.00	10.05	390.00	61.91
full vision	4	640.00	89.13	320.00	50.00	320.00	9.71	440.00	68.75
	5	580.00	99.06	250.00	43.10	330.00	9.26	380.00	65.52
	6	550.00	114.15	210.00	38.18	340.00	9.77	370.00	67.27
	7	610.00	106.79	290.00	47.54	320.00	11.20	370.00	60.66
	8	530.00	91.62	260.00	49.06	270.00	10.67	380.00	71.70
	9	600.00	95.33	300.00	50.00	300.00	9.74	460.00	76.67
	10	550.00	94.85	260.00	47.27	290.00	10.50	400.00	72.73
	11	580.00	107.09	240.00	41.38	340.00	9.35	400.00	68.97
	12	500.00	99.90	220.00	44.00	280.00	9.55	350.00	70.00
	13	530.00	96.65	270.00	50.94	260.00	8.56	370.00	69.81
	14	540.00	94.62	200.00	37.04	340.00	9.20	370.00	68.52
	15	560.00	111.54	90.00	16.07	470.00	7.45	430.00	76.79
	16	540.00	85.78	240.00	44.44	300.00	7.31	430.00	79.63
	17	550.00	87.65	290.00	52.73	260.00	8.21	410.00	74.55
	max	640.00	114.15	320.00	52.73	470.00	11.20	460.00	79.63
	mean	566.00	97.68	246.67	43.54	319.33	9.37	396.67	70.23
	SD	39.79	8.71	54.73	8.94	52.98	1.11	31.55	5.34

Participant	Trial no.	TMT (ms)	MV (cm/ms)	TMV (ms)	%TMV	DT (ms)	MA (cm)	TMA (ms)	%TMA
M10	3	650.00	86.69	240.00	36.92	410.00	8.76	460.00	70.77
full vision	4	680.00	78.36	280.00	41.18	400.00	7.31	490.00	72.06
	5	690.00	92.16	370.00	53.62	320.00	8.72	540.00	78.26
	6	660.00	82.53	310.00	46.97	350.00	7.32	480.00	72.73
	7	600.00	94.88	240.00	40.00	360.00	7.26	450.00	75.00
	8	680.00	72.78	330.00	48.53	350.00	7.46	460.00	67.65
	9	640.00	87.23	310.00	48.44	330.00	7.76	460.00	71.88
	10	660.00	97.08	280.00	42.42	380.00	8.64	500.00	75.76
	11	650.00	96.32	300.00	46.15	350.00	8.41	470.00	72.31
	12	630.00	92.77	280.00	44.44	350.00	7.75	490.00	77.78
	13	640.00	92.50	310.00	48.44	330.00	8.03	510.00	79.69
	14	740.00	89.11	340.00	45.95	400.00	8.73	520.00	70.27
	15	620.00	100.83	270.00	43.55	350.00	8.10	430.00	69.36
	16	610.00	106.26	290.00	47.54	320.00	9.68	420.00	68.85
	17	610.00	96.24	280.00	45.90	330.00	8.24	470.00	77.05
	max	740.00	106.26	370.00	53.62	410.00	9.68	540.00	79.69
	mean	650.67	91.05	295.33	45.34	355.33	8.14	476.67	73.29
	SD	36.74	8.63	35.02	4.11	29.49	0.69	32.66	3.74

Participant	Trial no.	TMT (ms)	MV (cm/ms)	TMV (ms)	%TMV	DT (ms)	MA (cm)	TMA (ms)	%TMA
M11	3	410.00	132.04	200.00	48.78	210.00	7.71	310.00	75.61
full vision	4	360.00	140.65	190.00	52.78	170.00	8.85	270.00	75.00
	5	480.00	116.07	230.00	47.92	250.00	7.75	380.00	79.17
	6	390.00	138.87	180.00	46.15	210.00	9.11	310.00	79.49
	7	380.00	153.74	160.00	42.11	220.00	8.56	290.00	76.32
	8	440.00	125.48	210.00	47.73	230.00	8.52	350.00	79.55
	9	380.00	116.38	160.00	42.11	220.00	9.28	300.00	78.95
	10	440.00	114.78	200.00	45.46	240.00	8.30	350.00	79.55
	11	450.00	126.16	170.00	37.78	280.00	8.59	290.00	64.44
	12	430.00	116.57	230.00	53.49	200.00	8.15	330.00	76.74
	13	410.00	126.69	180.00	43.90	230.00	8.53	330.00	80.49
	14	480.00	118.83	250.00	52.08	230.00	7.32	360.00	75.00
	18	430.00	115.14	180.00	41.86	250.00	7.35	340.00	79.07
	16	470.00	114.79	250.00	53.19	220.00	8.18	310.00	65.96
	17	460.00	115.82	230.00	50.00	230.00	7.64	370.00	80.44
	mean	427.33	124.80	201.33	47.02	226.00	8.26	326.00	76.38
	SD	38.07	11.86	30.67	4.82	25.01	0.61	32.25	4.93
	max	480.00	153.74	250.00	53.49	280.00	9.28	380.00	80.49

Participant	Trial no.	TMT (ms)	MV (cm/ms)	TMV (ms)	%TMV	DT (ms)	MA (cm)	TMA (ms)	%TMA
M12	3	740.00	88.54	300.00	40.54	440.00	9.87	550.00	74.32
full vision	4	720.00	88.26	320.00	44.44	400.00	11.29	540.00	75.00
	5	710.00	100.25	360.00	50.70	350.00	11.32	510.00	71.83
	6	680.00	89.55	260.00	38.24	420.00	10.93	500.00	73.53
	7	710.00	83.32	290.00	40.85	420.00	11.25	490.00	69.01
	8	680.00	86.58	310.00	45.59	370.00	10.97	510.00	75.00
	9	650.00	87.59	330.00	50.77	320.00	11.32	510.00	78.46
	18	690.00	91.46	370.00	53.62	320.00	10.58	540.00	78.26
	11	660.00	95.29	290.00	43.94	370.00	11.64	530.00	80.30
	12	700.00	84.83	310.00	44.29	390.00	10.84	570.00	81.43
	13	750.00	101.50	350.00	46.67	400.00	12.47	590.00	78.67
	14	720.00	86.38	240.00	33.33	480.00	11.32	540.00	75.00
	15	610.00	96.34	300.00	49.18	310.00	11.05	460.00	75.41
	19	620.00	95.32	310.00	50.00	310.00	11.13	490.00	79.03
	17	640.00	89.63	280.00	43.75	360.00	11.39	500.00	78.13
	mean	685.33	90.99	308.00	45.06	377.33	11.16	522.00	76.23
	SD	42.24	5.53	35.29	5.40	50.77	0.55	33.85	3.36
	max	750.00	101.50	370.00	53.62	480.00	12.47	590.00	81.43

Participant	Trial no.	TMT (ms)	MV (cm/ms)	TMV (ms)	%TMV	DT (ms)	MA (cm)	TMA (ms)	%TMA
O01	3	640.00	93.38	210.00	32.81	430.00	4.34	470.00	73.44
full vision	trial 4 ST=0, 19	600.00	102.63	240.00	40.00	360.00	5.35	370.00	61.67
	5	690.00	91.77	270.00	39.13	420.00	4.20	480.00	69.57
(Older)	6	670.00	82.40	250.00	37.31	420.00	4.86	470.00	70.15
	7	610.00	98.32	230.00	37.71	380.00	4.79	440.00	72.13
	8	650.00	76.50	270.00	41.54	380.00	4.33	490.00	75.39
	9	730.00	73.85	280.00	38.36	450.00	4.85	540.00	73.97
	10	580.00	100.91	250.00	43.10	330.00	4.62	440.00	75.86
	11	580.00	104.29	250.00	43.10	330.00	4.97	450.00	77.59
	12	560.00	108.41	230.00	41.07	330.00	4.91	410.00	73.21
	13	590.00	97.63	260.00	44.07	330.00	4.84	430.00	72.88
	14	740.00	100.51	320.00	43.24	420.00	5.12	510.00	68.92
	15	600.00	108.84	100.00	16.67	500.00	5.62	410.00	68.33
	16	580.00	99.23	250.00	43.10	330.00	5.38	400.00	68.97
	17	640.00	106.52	310.00	48.44	330.00	5.43	190.00	29.69
	mean	630.67	96.35	248.00	39.31	382.67	4.91	433.33	68.78
	SD	56.12	10.97	50.17	7.25	54.70	0.42	80.68	11.49
	max	740.00	108.84	320.00	48.44	500.00	5.62	540.00	77.59

Participant	Trial no.	TMT (ms)	MV (cm/ms)	TMV (ms)	%TMV	DT (ms)	MA (cm)	TMA (ms)	%TMA
O02	3	720.00	75.70	270.00	37.50	450.00	6.35	520.00	72.22
full vision	4	730.00	96.15	300.00	41.10	430.00	7.18	490.00	67.12
	5	710.00	87.45	310.00	43.66	400.00	6.42	480.00	67.61
	6	640.00	93.83	280.00	43.75	360.00	6.63	500.00	78.13
	7	610.00	97.63	210.00	34.43	400.00	6.08	450.00	73.77
	8	620.00	74.98	240.00	38.71	380.00	6.44	460.00	74.19
	9	670.00	72.10	240.00	35.82	430.00	5.95	510.00	76.12
	10	670.00	91.00	300.00	44.78	370.00	6.12	460.00	68.66
	11	620.00	87.67	250.00	40.32	370.00	7.01	470.00	75.81
	12	660.00	83.56	310.00	46.97	350.00	5.25	580.00	87.88
	13	660.00	84.29	220.00	33.33	440.00	5.69	510.00	77.27
	14	640.00	84.65	250.00	39.06	390.00	5.76	500.00	78.13
	15	640.00	91.19	270.00	42.19	370.00	6.34	500.00	78.13
	16	590.00	96.69	270.00	45.76	320.00	6.31	480.00	81.36
	17	640.00	93.21	260.00	40.63	380.00	6.07	520.00	81.25
	mean	654.67	87.34	265.33	40.53	389.33	6.24	495.33	75.84
	SD	40.51	8.14	31.14	4.11	36.15	0.49	32.26	5.59
	max	730.00	97.63	310.00	46.97	450.00	7.18	580.00	87.88

Participant	Trial no.	TMT (ms)	MV (cm/ms)	TMV (ms)	%TMV	DT (ms)	MA (cm)	TMA (ms)	%TMA
O03	3	540.00	93.70	220.00	40.74	320.00	8.54	380.00	70.37
full vision	4	550.00	105.69	240.00	43.64	310.00	9.10	330.00	60.00
	5	490.00	115.02	130.00	26.53	360.00	8.21	320.00	65.31
	6	490.00	108.97	200.00	40.82	290.00	8.97	330.00	67.35
	7	430.00	94.42	250.00	58.14	180.00	9.14	310.00	72.09
	8	470.00	109.60	130.00	27.66	340.00	9.30	330.00	70.21
	9	480.00	100.08	140.00	29.17	340.00	9.45	350.00	72.92
	10	460.00	85.65	140.00	30.44	320.00	9.45	280.00	60.87
	11	440.00	97.40	130.00	29.55	310.00	9.15	320.00	72.73
	12	490.00	114.75	100.00	20.41	390.00	9.30	340.00	69.39
	13	480.00	100.66	240.00	50.00	240.00	9.44	340.00	70.83
	14	450.00	107.65	130.00	28.89	320.00	8.73	350.00	77.78
	15	450.00	91.27	200.00	44.44	250.00	9.01	370.00	82.22
	16	470.00	103.29	140.00	29.79	330.00	8.96	370.00	78.72
	17	490.00	90.68	220.00	44.90	270.00	9.07	330.00	67.35
	mean	478.67	101.25	174.00	36.34	304.67	9.06	336.67	70.54
	SD	33.14	9.03	51.24	10.52	52.35	0.35	25.54	6.11
	max	550.00	115.02	250.00	58.14	390.00	9.45	380.00	82.22

Participant	Trial no.	TMT (ms)	MV (cm/ms)	TMV (ms)	%TMV	DT (ms)	MA (cm)	TMA (ms)	%TMA
O04	3	620.00	96.48	260.00	41.94	360.00	8.23	470.00	75.81
full vision	4	700.00	102.25	320.00	45.71	380.00	7.75	500.00	71.43
	5	630.00	102.77	320.00	50.79	310.00	7.60	510.00	80.95
	6	590.00	101.16	260.00	44.07	330.00	7.72	440.00	74.58
	7	720.00	91.46	320.00	44.44	400.00	7.84	520.00	72.22
	8	600.00	103.01	290.00	48.33	310.00	8.12	440.00	73.33
	9	670.00	105.86	290.00	43.28	380.00	7.83	520.00	77.61
	10	680.00	99.01	330.00	48.53	350.00	7.18	510.00	75.00
	11	670.00	112.96	250.00	37.31	420.00	7.21	490.00	73.13
	12	630.00	112.77	290.00	46.03	340.00	8.09	470.00	74.60
	13	580.00	109.71	290.00	50.00	290.00	7.66	420.00	72.41
	14	630.00	107.28	330.00	52.38	300.00	7.68	470.00	74.60
	15	560.00	109.20	310.00	55.36	250.00	7.44	460.00	82.14
	16	590.00	77.67	110.00	18.64	480.00	7.85	470.00	79.66
	17	580.00	90.39	280.00	48.28	300.00	6.71	450.00	77.59
	mean	630.00	101.46	283.33	45.01	346.67	7.66	476.00	75.67
	SD	48.40	9.51	54.47	8.55	58.76	0.40	31.35	3.27
	max	720.00	112.96	330.00	55.36	480.00	8.23	520.00	82.14

Participant	Trial no.	TMT (ms)	MV (cm/ms)	TMV (ms)	%TMV	DT (ms)	MA (cm)	TMA (ms)	%TMA
O05	3	640.00	83.22	290.00	45.31	350.00	7.74	510.00	79.69
full vision	4	600.00	89.19	350.00	58.33	250.00	8.21	480.00	80.00
	5	750.00	84.35	390.00	52.00	360.00	7.17	590.00	78.67
	6	630.00	90.42	120.00	19.05	510.00	8.45	150.00	23.81
	7	680.00	85.92	150.00	22.06	530.00	13.66	190.00	27.94
	8	600.00	98.24	120.00	20.00	480.00	12.05	190.00	31.67
	9	630.00	101.48	330.00	52.38	300.00	11.88	190.00	30.16
	10	640.00	101.21	130.00	20.31	510.00	13.44	220.00	34.38
	11	630.00	92.65	120.00	19.05	510.00	11.87	190.00	30.16
	12	530.00	97.23	310.00	58.49	220.00	11.00	230.00	43.40
	13	570.00	97.04	340.00	59.65	230.00	12.38	230.00	40.35
	14	600.00	88.07	330.00	55.00	270.00	12.88	200.00	33.33
	15	650.00	102.71	300.00	46.15	350.00	8.32	240.00	36.92
	16	620.00	96.56	360.00	58.07	260.00	10.71	190.00	30.65
	17	720.00	91.98	140.00	19.44	580.00	11.47	200.00	27.78
	mean	632.67	93.35	252.00	40.35	380.67	10.75	266.67	41.93
	SD	54.70	6.40	106.05	17.70	126.29	2.19	138.03	20.03
	max	750.00	102.71	390.00	59.65	580.00	13.66	590.00	80.00

Participant	Trial no.	TMT (ms)	MV (cm/ms)	TMV (ms)	%TMV	DT (ms)	MA (cm)	TMA (ms)	%TMA
O06	3	440.00	116.11	110.00	25.00	330.00	8.99	330.00	75.00
full vision	4	490.00	116.60	140.00	28.57	350.00	9.42	320.00	65.31
	5	410.00	135.85	120.00	29.27	290.00	9.77	300.00	73.17
	6	410.00	114.75	260.00	63.42	150.00	11.21	310.00	75.61
	7	460.00	129.60	120.00	26.09	340.00	8.43	320.00	69.57
	8	560.00	134.78	130.00	23.21	430.00	8.91	400.00	71.43
	9	410.00	123.57	110.00	26.83	300.00	9.38	300.00	73.17
	10	510.00	107.85	140.00	27.45	370.00	8.94	330.00	64.71
	11	470.00	114.67	120.00	25.53	350.00	9.35	320.00	68.09
	12	600.00	125.62	120.00	20.00	480.00	8.44	340.00	56.67
	13	510.00	90.45	100.00	19.61	410.00	7.86	130.00	25.49
	14	470.00	103.76	110.00	23.40	360.00	8.52	350.00	74.47
	15	390.00	140.78	110.00	28.21	280.00	8.49	310.00	79.49
	16	530.00	141.34	110.00	20.76	420.00	8.51	350.00	66.04
	17	590.00	109.26	180.00	30.51	410.00	7.20	420.00	71.19
	mean	483.33	120.33	132.00	27.86	351.33	8.89	322.00	67.29
	SD	66.62	14.58	40.39	10.40	79.00	0.91	63.16	12.84
	max	600.00	141.34	260.00	63.42	480.00	11.21	420.00	79.49

Participant	Trial no.	TMT (ms)	MV (cm/ms)	TMV (ms)	%TMV	DT (ms)	MA (cm)	TMA (ms)	%TMA
O07	3	860.00	95.36	350.00	40.70	510.00	8.32	580.00	67.44
full vision	4	960.00	73.27	460.00	47.92	500.00	7.05	770.00	80.21
	5	700.00	91.79	320.00	45.71	380.00	8.50	550.00	78.57
	6	880.00	82.48	430.00	48.86	450.00	7.76	700.00	79.55
	7	730.00	103.81	340.00	46.58	390.00	7.92	550.00	75.34
	8	810.00	83.54	420.00	51.85	390.00	6.92	640.00	79.01
	9	720.00	94.08	290.00	40.28	430.00	8.47	520.00	72.22
	10	840.00	87.64	320.00	38.10	520.00	8.22	600.00	71.43
	11	830.00	101.25	410.00	49.40	420.00	9.56	650.00	78.31
	12	850.00	89.78	320.00	37.65	530.00	8.27	620.00	72.94
	13	740.00	105.16	330.00	44.60	410.00	9.04	590.00	79.73
	14	810.00	93.25	370.00	45.68	440.00	8.98	600.00	74.07
	15	900.00	83.51	360.00	40.00	540.00	8.29	650.00	72.22
	16	870.00	83.88	370.00	42.53	500.00	7.78	670.00	77.01
	17	770.00	95.40	360.00	46.75	410.00	8.42	580.00	75.33
	mean	818.00	90.95	363.33	44.44	454.67	8.23	618.00	75.56
	SD	73.99	8.80	47.91	4.35	56.30	0.70	64.50	3.78
	max	960.00	105.16	460.00	51.85	540.00	9.56	770.00	80.21

Participant	Trial no.	TMT (ms)	MV (cm/ms)	TMV (ms)	%TMV	DT (ms)	MA (cm)	TMA (ms)	%TMA
O08	3	930.00	76.25	390.00	41.94	540.00	6.27	620.00	66.67
full vision	4	870.00	89.13	180.00	20.69	690.00	6.07	500.00	57.47
	5	870.00	102.74	330.00	37.93	540.00	6.98	510.00	58.62
	6	880.00	109.57	320.00	36.36	560.00	7.15	520.00	59.09
	7	760.00	103.36	270.00	35.53	490.00	6.76	480.00	63.16
	8	800.00	106.55	290.00	36.25	510.00	7.35	490.00	61.25
	9	650.00	108.09	270.00	41.54	380.00	7.64	510.00	78.46
	10	540.00	117.13	230.00	42.59	310.00	8.53	380.00	70.37
	11	690.00	111.78	280.00	40.58	410.00	7.71	480.00	69.57
	12	590.00	113.37	230.00	38.98	360.00	9.37	430.00	72.88
	13	690.00	89.26	380.00	55.07	310.00	7.62	530.00	76.81
	14	600.00	108.20	240.00	40.00	360.00	9.91	430.00	71.67
	15	580.00	120.78	240.00	41.38	340.00	8.92	410.00	70.69
	16	590.00	112.75	200.00	33.90	390.00	9.38	400.00	67.80
	17	570.00	114.29	210.00	36.84	360.00	8.61	380.00	66.67
	mean	707.33	105.55	270.67	38.64	436.67	7.89	471.33	67.41
	SD	133.55	12.05	62.50	7.01	111.53	1.18	66.10	6.47
	max	930.00	120.78	390.00	55.07	690.00	9.91	620.00	78.46

Participant	Trial no.	TMT (ms)	MV (cm/ms)	TMV (ms)	%TMV	DT (ms)	MA (cm)	TMA (ms)	%TMA
O09	3	570.00	98.03	110.00	19.30	460.00	7.09	370.00	64.91
full vision	4	620.00	80.91	260.00	41.94	360.00	7.27	410.00	66.13
	5	570.00	80.53	210.00	36.84	360.00	7.22	350.00	61.40
	6	530.00	78.10	220.00	41.51	310.00	7.29	310.00	58.49
	7	560.00	89.06	230.00	41.07	330.00	7.36	330.00	58.93
	8	580.00	85.83	240.00	41.38	340.00	7.60	410.00	70.69
	9	600.00	88.87	250.00	41.67	350.00	7.12	420.00	70.00
	10	550.00	92.87	220.00	40.00	330.00	6.64	380.00	69.09
	11	580.00	98.26	290.00	50.00	290.00	7.64	380.00	65.52
	12	610.00	88.89	270.00	44.26	340.00	6.90	400.00	65.57
	13	560.00	95.72	250.00	44.64	310.00	6.88	390.00	69.64
	14	600.00	88.89	250.00	41.67	350.00	6.74	400.00	66.67
	15	590.00	84.25	210.00	35.59	380.00	6.60	350.00	59.32
	16	620.00	89.14	280.00	45.16	340.00	6.47	420.00	67.74
	17	630.00	94.57	250.00	39.68	380.00	6.77	450.00	71.43
	mean	584.67	88.93	236.00	40.31	348.67	7.04	384.67	65.70
	SD	28.75	6.25	42.39	6.75	39.62	0.36	37.58	4.36
	max	630.00	98.26	290.00	50.00	460.00	7.64	450.00	71.43

Participant	Trial no.	TMT (ms)	MV (cm/ms)	TMV (ms)	%TMV	DT (ms)	MA (cm)	TMA (ms)	%TMA
O10	3	980.00	86.48	110.00	11.22	870.00	8.11	600.00	61.22
full vision	4	980.00	69.36	160.00	16.33	820.00	6.82	660.00	67.35
	5	740.00	97.90	180.00	24.32	560.00	8.54	470.00	63.51
	6	750.00	100.24	130.00	17.33	620.00	8.87	550.00	73.33
	7	850.00	100.46	180.00	21.18	670.00	7.64	560.00	65.88
	8	740.00	91.47	350.00	47.30	390.00	8.70	550.00	74.32
	9	700.00	84.78	290.00	41.43	410.00	8.34	570.00	81.43
	10	660.00	99.26	260.00	39.39	400.00	7.83	460.00	69.70
	11	710.00	90.33	290.00	40.85	420.00	8.19	510.00	71.83
	12	730.00	90.87	310.00	42.47	420.00	8.43	530.00	72.60
	13	640.00	117.13	100.00	15.63	540.00	8.45	480.00	75.00
	14	660.00	88.62	280.00	42.42	380.00	8.00	500.00	75.76
	15	800.00	91.94	190.00	23.75	610.00	8.13	510.00	63.75
	16	720.00	117.28	170.00	23.61	550.00	7.10	520.00	72.22
	17	620.00	84.44	290.00	46.77	330.00	7.99	510.00	82.26
	mean	752.00	94.04	219.33	30.27	532.67	8.08	532.00	71.34
	SD	109.88	12.26	80.04	12.88	163.16	0.56	52.26	6.20
	max	980.00	117.28	350.00	47.30	870.00	8.87	660.00	82.26

Participant	Trial no.	TMT (ms)	MV (cm/ms)	TMV (ms)	%TMV	DT (ms)	MA (cm)	TMA (ms)	%TMA
O11	3	470.00	132.80	190.00	40.43	280.00	11.60	330.00	70.21
full vision	4	400.00	135.33	150.00	37.50	250.00	11.35	270.00	67.50
	5	420.00	131.75	180.00	42.86	240.00	11.90	300.00	71.43
	6	530.00	119.67	190.00	35.85	340.00	9.68	360.00	67.93
	7	570.00	141.71	190.00	33.33	380.00	9.79	380.00	66.67
	8	550.00	115.71	180.00	32.73	370.00	9.62	340.00	61.82
	9	450.00	132.95	210.00	46.67	240.00	11.23	340.00	75.56
	10	480.00	125.17	200.00	41.67	280.00	9.60	320.00	66.67
	11	610.00	119.97	260.00	42.62	350.00	9.74	400.00	65.57
	12	560.00	112.05	230.00	41.07	330.00	9.33	410.00	73.21
	13	500.00	102.30	180.00	36.00	320.00	9.44	350.00	70.00
	14	510.00	105.64	200.00	39.22	310.00	9.67	350.00	68.63
	15	500.00	105.59	180.00	36.00	320.00	9.61	290.00	58.00
	16	570.00	120.25	220.00	38.60	350.00	10.03	330.00	57.90
	17	460.00	114.51	160.00	34.78	300.00	9.80	290.00	63.04
	mean	505.33	121.03	194.67	38.62	310.67	10.16	337.33	66.94
	SD	59.98	12.03	27.48	3.94	45.11	0.87	40.26	5.09
	max	610.00	141.71	260.00	46.67	380.00	11.90	410.00	75.56

Participant	Trial no.	TMT (ms)	MV (cm/ms)	TMV (ms)	%TMV	DT (ms)	MA (cm)	TMA (ms)	%TMA
O12	3	490.00	100.44	230.00	46.94	260.00	9.45	360.00	73.47
full vision	4	470.00	109.02	210.00	44.68	260.00	9.39	340.00	72.34
	5	450.00	115.54	180.00	40.00	270.00	8.97	310.00	68.89
	6	420.00	106.48	240.00	57.14	180.00	9.59	300.00	71.43
	18	390.00	104.61	190.00	48.72	200.00	8.68	280.00	71.80
	8	460.00	109.23	220.00	47.83	240.00	9.26	330.00	71.74
	9	480.00	95.75	270.00	56.25	210.00	9.33	340.00	70.83
	10	430.00	118.34	230.00	53.49	200.00	9.79	320.00	74.42
	11	460.00	102.46	290.00	63.04	170.00	9.71	340.00	73.91
	12	470.00	110.63	310.00	65.96	160.00	9.21	360.00	76.60
	13	470.00	110.72	230.00	48.94	240.00	9.35	350.00	74.47
	14	440.00	138.64	210.00	47.73	230.00	11.47	290.00	65.91
	15	460.00	107.99	220.00	47.83	240.00	10.30	320.00	69.57
	16	430.00	110.28	190.00	44.19	240.00	9.84	280.00	65.12
	17	480.00	105.78	250.00	52.08	230.00	9.54	350.00	72.92
	mean	453.33	109.73	231.33	50.99	222.00	9.59	324.67	71.56
	SD	26.90	9.77	36.62	7.10	33.85	0.64	27.48	3.15
	max	490.00	138.64	310.00	65.96	270.00	11.47	360.00	76.60
