# Supplementary material for: Age-related changes in reach-to-grasp movements with partial visual occlusion
Source: PLoS One. 2019 Aug 28;14(8):e0221320. doi: 10.1371/journal.pone.0221320 (PMC6713340; doi:10.1371/journal.pone.0221320)
Supplement: S4 Table — Raw data of individual participant in visual occlusion condition for younger, middle-aged and older groups. (PDF) [file pone.0221320.s004.pdf]

Table S4. Raw data of individual participant in visual occlusion condition in younger, middle-aged and older groups

Participant	Trial no.	TMT (ms)	MV (cm/ms)	TMV (ms)	%TMV	DT (ms)	MA (cm)	TMA (ms)	%TMA
Y01	3	400.00	139.98	190.00	47.50	210.00	8.15	300.00	75.00
visual occlusion	4	400.00	141.15	160.00	40.00	240.00	8.27	250.00	62.50
	5	350.00	135.63	140.00	40.00	210.00	8.50	230.00	65.71
(Younger)	6	490.00	156.45	160.00	32.65	330.00	8.08	240.00	48.98
	7	420.00	140.63	150.00	35.71	270.00	10.49	240.00	57.14
	8	380.00	139.58	130.00	34.21	250.00	10.20	270.00	71.05
	9	460.00	143.75	150.00	32.61	310.00	10.16	260.00	56.52
	10	380.00	148.15	180.00	47.37	200.00	10.49	240.00	63.16
	11	440.00	130.45	160.00	36.36	280.00	10.42	240.00	54.55
	12	450.00	149.38	160.00	35.56	290.00	10.23	250.00	55.56
	13	390.00	135.78	150.00	38.46	240.00	9.97	260.00	66.67
	14	370.00	145.86	140.00	37.84	230.00	9.82	260.00	70.27
	15	370.00	153.45	140.00	37.84	230.00	9.76	240.00	64.87
	16	410.00	140.03	150.00	36.59	260.00	11.09	250.00	60.98
	17	370.00	153.94	130.00	35.14	240.00	11.26	240.00	64.87
	max	490.00	156.45	190.00	47.50	330.00	11.26	300.00	75.00
	mean	405.33	143.61	152.67	37.86	252.67	9.79	251.33	62.52
	SD	39.62	7.48	16.68	4.49	37.51	1.05	17.27	7.02

Participant	Trial no.	TMT (ms)	MV (cm/ms)	TMV (ms)	%TMV	DT (ms)	MA (cm)	TMA (ms)	%TMA
Y02	3	380.00	143.84	110.00	28.95	270.00	10.89	240.00	63.16
visual occlusion	4	340.00	151.28	50.00	14.71	290.00	10.93	180.00	52.94
	5	380.00	185.90	100.00	26.32	280.00	10.21	230.00	60.53
	6	390.00	161.78	110.00	28.21	280.00	11.65	250.00	64.10
	7	390.00	155.04	100.00	25.64	290.00	10.83	250.00	64.10
	8	400.00	171.53	110.00	27.50	290.00	12.00	230.00	57.50
	9	400.00	160.45	130.00	32.50	270.00	10.83	260.00	65.00
	10	390.00	152.39	130.00	33.33	260.00	10.79	260.00	66.67
	11	400.00	185.82	90.00	22.50	310.00	10.63	240.00	60.00
	12	320.00	209.18	90.00	28.13	230.00	11.27	160.00	50.00
	13	370.00	166.04	110.00	29.73	260.00	10.90	220.00	59.46
	14	390.00	138.61	80.00	20.51	310.00	9.32	260.00	66.67
	15	390.00	137.71	110.00	28.21	280.00	10.12	260.00	66.67
	16	380.00	156.21	100.00	26.32	280.00	11.04	260.00	68.42
	17	370.00	139.49	110.00	29.73	260.00	10.05	250.00	67.57
	max	400.00	209.18	130.00	33.33	310.00	12.00	260.00	68.42
	mean	379.33	161.02	102.00	26.82	277.33	10.76	236.67	62.19
	SD	22.51	20.22	19.71	4.70	20.52	0.66	30.16	5.45

Participant	Trial no.	TMT (ms)	MV (cm/ms)	TMV (ms)	%TMV	DT (ms)	MA (cm)	TMA (ms)	%TMA
Y03	3	460.00	100.67	260.00	56.52	200.00	8.73	320.00	69.57
visual occlusion	4	490.00	107.01	120.00	24.49	370.00	9.53	310.00	63.27
	5	440.00	117.66	130.00	29.55	310.00	9.72	270.00	61.36
	6	460.00	110.77	240.00	52.17	220.00	10.45	280.00	60.87
	7	420.00	114.94	190.00	45.24	230.00	10.05	270.00	64.29
	8	480.00	118.49	120.00	25.00	360.00	9.67	330.00	68.75
	9	420.00	134.24	130.00	30.95	290.00	11.33	240.00	57.14
	10	480.00	113.84	220.00	45.83	260.00	11.79	280.00	58.33
	11	480.00	117.41	210.00	43.75	270.00	10.82	290.00	60.42
	12	490.00	115.30	190.00	38.78	300.00	12.03	270.00	55.10
	13	490.00	119.18	210.00	42.86	280.00	11.47	300.00	61.22
	14	430.00	123.69	130.00	30.23	300.00	11.86	270.00	62.79
	15	470.00	124.86	170.00	36.17	300.00	10.45	280.00	59.57
	16	390.00	145.60	110.00	28.21	280.00	12.13	250.00	64.10
	17	420.00	133.59	120.00	28.57	300.00	11.49	240.00	57.14
	max	490.00	145.60	260.00	56.52	370.00	12.13	330.00	69.57
	mean	454.67	119.82	170.00	37.22	284.67	10.77	280.00	61.60
	SD	32.26	11.36	50.28	10.10	46.12	1.06	26.73	4.06

Participant	Trial no.	TMT (ms)	MV (cm/ms)	TMV (ms)	%TMV	DT (ms)	MA (cm)	TMA (ms)	%TMA
Y04 visual occlusion	3	420.00	111.81	140.00	33.33	280.00	10.50	330.00	78.57
	2	460.00	94.14	170.00	36.96	290.00	9.46	360.00	78.26
	18	380.00	129.96	130.00	34.21	250.00	10.96	240.00	63.16
	19	380.00	111.86	230.00	60.53	150.00	10.56	270.00	71.05
	7	410.00	133.93	150.00	36.59	260.00	11.15	250.00	60.98
	8	410.00	116.21	170.00	41.46	240.00	11.23	300.00	73.17
	9	380.00	114.55	140.00	36.84	240.00	10.98	250.00	65.79
	10	400.00	125.93	120.00	30.00	280.00	10.02	310.00	77.50
	11	370.00	114.34	150.00	40.54	220.00	11.12	260.00	70.27
	12	360.00	113.16	140.00	38.89	220.00	10.98	260.00	72.22
	13	380.00	122.64	130.00	34.21	250.00	10.87	280.00	73.68
	14	370.00	115.67	120.00	32.43	250.00	11.45	240.00	64.87
	15	360.00	118.26	130.00	36.11	230.00	10.77	260.00	72.22
	16	360.00	117.41	170.00	47.22	190.00	11.56	260.00	72.22
	20	410.00	111.32	210.00	51.22	200.00	10.75	280.00	68.29
	max	460.00	133.93	230.00	60.53	290.00	11.56	360.00	78.57
	mean	390.00	116.75	153.33	39.37	236.67	10.82	276.67	70.82
	SD	28.03	9.28	32.00	8.07	37.16	0.54	34.57	5.36

Participant	Trial no.	TMT (ms)	MV (cm/ms)	TMV (ms)	%TMV	DT (ms)	MA (cm)	TMA (ms)	%TMA
Y05 visual occlusion	18	390.00	132.56	210.00	53.85	180.00	10.87	280.00	71.80
	4	350.00	134.02	140.00	40.00	210.00	9.91	260.00	74.29
	5	370.00	130.84	170.00	45.95	200.00	9.70	280.00	75.68
	6	380.00	155.95	150.00	39.47	230.00	9.90	270.00	71.05
	7	340.00	138.89	160.00	47.06	180.00	9.96	250.00	73.53
	8	360.00	135.68	160.00	44.44	200.00	9.83	260.00	72.22
	9	380.00	134.20	150.00	39.47	230.00	10.08	280.00	73.68
	10	360.00	134.28	170.00	47.22	190.00	9.73	250.00	69.44
	11	370.00	131.18	160.00	43.24	210.00	9.70	260.00	70.27
	12	330.00	145.65	150.00	45.46	180.00	9.93	250.00	75.76
	13	370.00	134.34	150.00	40.54	220.00	9.67	270.00	72.97
	14	360.00	120.48	140.00	38.89	220.00	10.14	250.00	69.44
	15	330.00	148.00	150.00	45.46	180.00	10.30	230.00	69.70
	16	370.00	139.12	160.00	43.24	210.00	10.11	250.00	67.57
	17	380.00	147.09	170.00	44.74	210.00	11.22	260.00	68.42
	max	390.00	155.95	210.00	53.85	230.00	11.22	280.00	75.76
	mean	362.67	137.49	159.33	43.94	203.33	10.07	260.00	71.72
	SD	18.31	8.70	17.10	3.98	17.99	0.44	14.14	2.57

Participant	Trial no.	TMT (ms)	MV (cm/ms)	TMV (ms)	%TMV	DT (ms)	MA (cm)	TMA (ms)	%TMA
Y06 visual occlusion	3	370.00	173.34	100.00	27.03	270.00	12.45	210.00	56.76
	4	390.00	159.88	90.00	23.08	300.00	11.59	260.00	66.67
	5	360.00	185.66	100.00	27.78	260.00	12.38	210.00	58.33
	6	350.00	160.15	110.00	31.43	240.00	13.03	220.00	62.86
	7	340.00	192.41	110.00	32.35	230.00	12.94	190.00	55.88
	8	320.00	168.03	110.00	34.38	210.00	12.36	220.00	68.75
	9	380.00	140.73	120.00	31.58	260.00	11.09	250.00	65.79
	10	370.00	182.79	90.00	24.32	280.00	12.67	210.00	56.76
	11	300.00	199.69	90.00	30.00	210.00	13.38	190.00	63.33
	12	380.00	164.68	190.00	50.00	190.00	14.10	230.00	60.53
	13	340.00	187.26	100.00	29.41	240.00	11.72	230.00	67.65
	14	390.00	175.73	100.00	25.64	290.00	11.70	210.00	53.85
	15	420.00	154.16	90.00	21.43	330.00	9.60	280.00	66.67
	16	330.00	188.69	90.00	27.27	240.00	12.25	220.00	66.67
	17	450.00	163.84	90.00	20.00	360.00	11.79	240.00	53.33
	max	450.00	199.69	190.00	50.00	360.00	14.10	280.00	68.75
	mean	366.00	173.14	105.33	29.05	260.67	12.20	224.67	61.59
	SD	38.69	16.29	25.32	7.12	46.21	1.06	24.75	5.39

Participant	Trial no.	TMT (ms)	MV (cm/ms)	TMV (ms)	%TMV	DT (ms)	MA (cm)	TMA (ms)	%TMA
Y07	3	560.00	90.08	130.00	23.21	430.00	9.24	390.00	69.64
visual occlusion	4	500.00	99.39	270.00	54.00	230.00	9.86	340.00	68.00
	5	480.00	101.10	120.00	25.00	360.00	10.18	270.00	56.25
	6	520.00	115.74	120.00	23.08	400.00	7.90	400.00	76.92
	7	500.00	109.30	120.00	24.00	380.00	11.15	360.00	72.00
	8	490.00	121.66	120.00	24.49	370.00	9.62	350.00	71.43
	9	480.00	105.86	290.00	60.42	190.00	10.54	360.00	75.00
	10	480.00	105.89	280.00	58.33	200.00	9.21	350.00	72.92
	11	520.00	102.47	290.00	55.77	230.00	9.26	380.00	73.08
	12	490.00	113.84	120.00	24.49	370.00	9.38	290.00	59.18
	13	460.00	113.30	130.00	28.26	330.00	8.55	300.00	65.22
	14	460.00	117.25	130.00	28.26	330.00	9.24	320.00	69.57
	15	500.00	106.48	150.00	30.00	350.00	8.49	310.00	62.00
	16	490.00	105.03	110.00	22.45	380.00	8.96	340.00	69.39
	17	530.00	118.92	140.00	26.42	390.00	7.18	310.00	58.49
	max	560.00	121.66	290.00	60.42	430.00	11.15	400.00	76.92
	mean	497.33	108.42	168.00	33.88	329.33	9.25	338.00	67.94
	SD	26.58	8.48	72.23	14.72	77.69	1.00	37.64	6.34

Participant	Trial no.	TMT (ms)	MV (cm/ms)	TMV (ms)	%TMV	DT (ms)	MA (cm)	TMA (ms)	%TMA
Y08	3	550.00	110.00	130.00	23.64	420.00	9.13	360.00	65.46
visual occlusion	4	490.00	110.39	110.00	22.45	380.00	9.24	310.00	63.27
	18	400.00	119.35	190.00	47.50	210.00	8.61	270.00	67.50
	6	560.00	143.40	130.00	23.21	430.00	7.09	370.00	66.07
	7	550.00	112.51	280.00	50.91	270.00	7.97	370.00	67.27
	8	540.00	90.02	120.00	22.22	420.00	8.11	370.00	68.52
	9	440.00	122.59	230.00	52.27	210.00	8.86	320.00	72.73
	10	480.00	118.91	250.00	52.08	230.00	8.94	350.00	72.92
	11	440.00	135.18	240.00	54.55	200.00	9.02	320.00	72.73
	12	500.00	99.70	130.00	26.00	370.00	8.77	340.00	68.00
	13	410.00	110.00	240.00	58.54	170.00	8.80	290.00	70.73
	14	430.00	111.50	110.00	25.58	320.00	8.87	290.00	67.44
	15	410.00	122.24	210.00	51.22	200.00	8.07	300.00	73.17
	16	450.00	133.69	180.00	40.00	270.00	8.60	280.00	62.22
	17	410.00	120.51	200.00	48.78	210.00	9.04	270.00	65.85
	max	560.00	143.40	280.00	58.54	430.00	9.24	370.00	73.17
	mean	470.67	117.33	183.33	39.93	287.33	8.61	320.67	68.26
	SD	57.75	13.64	57.78	14.16	93.69	0.57	37.12	3.53

Participant	Trial no.	TMT (ms)	MV (cm/ms)	TMV (ms)	%TMV	DT (ms)	MA (cm)	TMA (ms)	%TMA
Y09	3	550.00	100.19	100.00	18.18	450.00	7.71	370.00	67.27
visual occlusion	4	540.00	95.04	240.00	44.44	300.00	8.09	390.00	72.22
	5	500.00	122.06	230.00	46.00	270.00	8.54	340.00	68.00
	6	490.00	115.44	220.00	44.90	270.00	8.41	360.00	73.47
	7	510.00	112.42	240.00	47.06	270.00	8.39	370.00	72.55
	8	490.00	112.52	260.00	53.06	230.00	8.97	340.00	69.39
	9	510.00	105.72	210.00	41.18	300.00	8.38	360.00	70.59
	10	500.00	98.87	250.00	50.00	250.00	8.42	370.00	74.00
	11	490.00	114.49	220.00	44.90	270.00	8.17	340.00	69.39
	12	510.00	108.76	240.00	47.06	270.00	7.79	350.00	68.63
	13	490.00	116.19	240.00	48.98	250.00	8.28	330.00	67.35
	14	480.00	111.61	90.00	18.75	390.00	8.11	340.00	70.83
	15	500.00	115.19	250.00	50.00	250.00	8.19	360.00	72.00
	16	530.00	111.32	90.00	16.98	440.00	7.78	330.00	62.26
	17	520.00	110.45	90.00	17.31	430.00	7.39	330.00	63.46
	max	550.00	122.06	260.00	53.06	450.00	8.97	390.00	74.00
	mean	507.33	110.02	198.00	39.25	309.33	8.17	352.00	69.43
	SD	20.17	7.28	67.10	13.68	76.85	0.39	18.21	3.42

Participant	Trial no.	TMT (ms)	MV (cm/ms)	TMV (ms)	%TMV	DT (ms)	MA (cm)	TMA (ms)	%TMA
Y10 visual occlusion	3	440.00	119.79	90.00	20.46	350.00	11.63	300.00	68.18
	4	430.00	133.08	80.00	18.61	350.00	12.14	290.00	67.44
	5	380.00	159.96	80.00	21.05	300.00	13.19	270.00	71.05
	6	450.00	155.71	90.00	20.00	360.00	13.24	340.00	75.56
	7	440.00	122.75	90.00	20.46	350.00	12.66	300.00	68.18
	8	430.00	112.66	200.00	46.51	230.00	11.84	310.00	72.09
	9	450.00	132.59	150.00	33.33	300.00	11.77	300.00	66.67
	10	460.00	123.56	100.00	21.74	360.00	12.15	330.00	71.74
	11	450.00	127.45	80.00	17.78	370.00	12.22	280.00	62.22
	12	440.00	126.64	80.00	18.18	360.00	12.12	290.00	65.91
	13	410.00	145.04	80.00	19.51	330.00	12.98	300.00	73.17
	14	420.00	136.49	80.00	19.05	340.00	12.00	240.00	57.14
	15	400.00	138.87	80.00	20.00	320.00	11.42	270.00	67.50
	16	480.00	128.96	90.00	18.75	390.00	12.06	290.00	60.42
	17	440.00	150.48	70.00	15.91	370.00	13.11	290.00	65.91
	max	480.00	159.96	200.00	46.51	390.00	13.24	340.00	75.56
	mean	434.67	134.27	96.00	22.09	338.67	12.30	293.33	67.55
	SD	24.75	13.59	34.18	7.79	39.25	0.59	24.10	4.93

Participant	Trial no.	TMT (ms)	MV (cm/ms)	TMV (ms)	%TMV	DT (ms)	MA (cm)	TMA (ms)	%TMA
Y11 visual occlusion	3	350.00	169.25	230.00	65.71	120.00	12.53	250.00	71.43
	4	360.00	164.04	210.00	58.33	150.00	11.32	260.00	72.22
	5	340.00	146.76	200.00	58.82	140.00	10.75	240.00	70.59
	6	340.00	151.63	200.00	58.82	140.00	10.72	250.00	73.53
	7	400.00	149.60	260.00	65.00	140.00	10.57	310.00	77.50
	8	380.00	158.36	210.00	55.26	170.00	9.92	270.00	71.05
	9	370.00	159.90	220.00	59.46	150.00	10.46	260.00	70.27
	10	350.00	139.88	190.00	54.29	160.00	9.77	270.00	77.14
	11	320.00	182.00	180.00	56.25	140.00	12.11	220.00	68.75
	12	400.00	149.62	240.00	60.00	160.00	9.79	290.00	72.50
	13	350.00	135.84	240.00	68.57	110.00	10.33	260.00	74.29
	14	370.00	173.42	250.00	67.57	120.00	11.51	270.00	72.97
	15	310.00	173.39	190.00	61.29	120.00	11.01	210.00	67.74
	16	350.00	157.58	230.00	65.71	120.00	10.05	250.00	71.43
	17	330.00	184.69	190.00	57.58	140.00	10.55	220.00	66.67
	mean	354.67	159.73	216.00	60.84	138.67	10.76	255.33	71.87
	SD	26.15	14.67	24.73	4.57	17.67	0.82	26.42	3.03
	max	400.00	184.69	260.00	68.57	170.00	12.53	310.00	77.50

Participant	Trial no.	TMT (ms)	MV (cm/ms)	TMV (ms)	%TMV	DT (ms)	MA (cm)	TMA (ms)	%TMA
Y12 visual occlusion	3	440.00	125.83	170.00	38.64	270.00	11.22	290.00	65.91
	4	420.00	111.61	150.00	35.71	270.00	10.34	290.00	69.05
	5	420.00	127.26	160.00	38.10	260.00	9.93	290.00	69.05
	6	420.00	98.83	220.00	52.38	200.00	9.88	290.00	69.05
	2	410.00	125.44	140.00	34.15	270.00	10.29	270.00	65.85
	8	420.00	127.25	220.00	52.38	200.00	10.81	280.00	66.67
	9	460.00	112.89	170.00	36.96	290.00	11.29	310.00	67.39
	10	430.00	135.57	150.00	34.88	280.00	10.46	260.00	60.47
	11	430.00	114.33	210.00	48.84	220.00	10.69	290.00	67.44
	18	420.00	132.38	210.00	50.00	210.00	11.80	270.00	64.29
	19	390.00	127.35	130.00	33.33	260.00	11.15	260.00	66.67
	14	490.00	109.51	230.00	46.94	260.00	11.06	330.00	67.35
	15	440.00	106.69	140.00	31.82	300.00	12.14	290.00	65.91
	16	390.00	121.56	190.00	48.72	200.00	11.56	250.00	64.10
	17	400.00	124.45	140.00	35.00	260.00	11.46	270.00	67.50
	mean	425.33	120.06	175.33	41.19	250.00	10.94	282.67	66.45
	SD	25.88	10.45	34.82	7.64	34.43	0.67	20.52	2.25
	max	490.00	135.57	230.00	52.38	300.00	12.14	330.00	69.05

Participant	Trial no.	TMT (ms)	MV (cm/ms)	TMV (ms)	%TMV	DT (ms)	MA (cm)	TMA (ms)	%TMA
M01	3	760.00	96.75	380.00	50.00	380.00	7.95	560.00	73.68
visual occlusion	4	670.00	81.37	340.00	50.75	330.00	9.14	470.00	70.15
	5	630.00	98.91	300.00	47.62	330.00	9.60	470.00	74.60
(Middle-aged)	6	610.00	87.00	320.00	52.46	290.00	8.82	460.00	75.41
	7	620.00	95.51	290.00	46.77	330.00	8.39	460.00	74.19
	8	700.00	75.06	330.00	47.14	370.00	6.72	500.00	71.43
	9	690.00	75.35	340.00	49.28	350.00	7.04	550.00	79.71
	10	620.00	87.85	320.00	51.61	300.00	8.09	460.00	74.19
	11	630.00	91.56	270.00	42.86	360.00	9.04	470.00	74.60
	12	690.00	86.16	370.00	53.62	320.00	7.03	500.00	72.46
	13	650.00	87.25	310.00	47.69	340.00	7.30	440.00	67.69
	14	580.00	87.47	270.00	46.55	310.00	6.96	430.00	74.14
	15	650.00	88.86	330.00	50.77	320.00	8.09	450.00	69.23
	16	570.00	102.78	270.00	47.37	300.00	8.04	390.00	68.42
	17	540.00	102.46	280.00	51.85	260.00	9.31	410.00	75.93
	max	760.00	102.78	380.00	53.62	380.00	9.60	560.00	79.71
	mean	640.67	89.62	314.67	49.09	326.00	8.10	468.00	73.06
	SD	56.37	8.58	35.02	2.85	31.80	0.94	45.86	3.20

Participant	Trial no.	TMT (ms)	MV (cm/ms)	TMV (ms)	%TMV	DT (ms)	MA (cm)	TMA (ms)	%TMA
M02	3	700.00	76.45	100.00	14.29	600.00	7.48	470.00	67.14
visual occlusion	4	490.00	92.67	220.00	44.90	270.00	7.36	330.00	67.35
	5	500.00	90.25	240.00	48.00	260.00	6.93	360.00	72.00
	6	620.00	92.62	270.00	43.55	350.00	7.51	390.00	62.90
	7	600.00	95.54	260.00	43.33	340.00	7.08	390.00	65.00
	8	620.00	94.95	280.00	45.16	340.00	7.82	430.00	69.36
	9	540.00	88.68	270.00	50.00	270.00	6.82	390.00	72.22
	10	520.00	101.64	240.00	46.15	280.00	7.17	360.00	69.23
	11	590.00	90.57	280.00	47.46	310.00	6.01	400.00	67.80
	12	670.00	79.71	300.00	44.78	370.00	5.63	470.00	70.15
	13	470.00	114.19	100.00	21.28	370.00	7.52	320.00	68.09
	14	510.00	104.13	110.00	21.57	400.00	7.16	360.00	70.59
	15	520.00	104.43	240.00	46.15	280.00	7.43	360.00	69.23
	16	420.00	109.12	100.00	23.81	320.00	9.35	290.00	69.05
	17	510.00	109.46	110.00	21.57	400.00	7.54	360.00	70.59
	max	700.00	114.19	300.00	50.00	600.00	9.35	470.00	72.22
	mean	552.00	96.29	208.00	37.47	344.00	7.25	378.67	68.71
	SD	78.03	10.82	78.67	12.68	85.00	0.83	50.41	2.48

Participant	Trial no.	TMT (ms)	MV (cm/ms)	TMV (ms)	%TMV	DT (ms)	MA (cm)	TMA (ms)	%TMA
M03	3	640.00	103.52	230.00	35.94	410.00	7.93	430.00	67.19
visual occlusion	4	590.00	102.20	260.00	44.07	330.00	8.31	420.00	71.19
	5	660.00	101.78	270.00	40.91	390.00	8.45	460.00	69.70
	18	520.00	104.69	210.00	40.39	310.00	8.49	390.00	75.00
	7	690.00	86.26	100.00	14.49	590.00	7.26	460.00	66.67
	8	580.00	96.15	230.00	39.66	350.00	7.27	430.00	74.14
	9	690.00	101.61	290.00	42.03	400.00	7.82	480.00	69.57
	10	630.00	101.99	110.00	17.46	520.00	6.94	490.00	77.78
	11	560.00	101.58	180.00	32.14	380.00	7.99	390.00	69.64
	12	570.00	94.34	160.00	28.07	410.00	8.12	290.00	50.88
	13	620.00	91.55	220.00	35.48	400.00	7.26	470.00	75.81
	14	600.00	103.81	210.00	35.00	390.00	8.05	400.00	66.67
	15	620.00	92.70	220.00	35.48	400.00	6.94	420.00	67.74
	16	630.00	91.17	260.00	41.27	370.00	7.25	500.00	79.37
	17	510.00	100.48	90.00	17.65	420.00	7.39	320.00	62.75
	max	690.00	104.69	290.00	44.07	590.00	8.49	500.00	79.37
	mean	607.33	98.26	202.67	33.34	404.67	7.70	423.33	69.60
	SD	53.91	5.72	62.73	9.64	69.58	0.54	59.60	6.95

Participant	Trial no.	TMT (ms)	MV (cm/ms)	TMV (ms)	%TMV	DT (ms)	MA (cm)	TMA (ms)	%TMA
M04 visual occlusion	3	630.00	90.95	260.00	41.27	370.00	8.27	430.00	68.25
	4	610.00	87.29	290.00	47.54	320.00	8.06	440.00	72.13
	5	660.00	83.78	270.00	40.91	390.00	7.17	470.00	71.21
	6	640.00	80.68	300.00	46.88	340.00	8.00	480.00	75.00
	7	640.00	77.18	240.00	37.50	400.00	6.50	470.00	73.44
	8	570.00	89.03	250.00	43.86	320.00	9.37	400.00	70.18
	9	620.00	77.13	280.00	45.16	340.00	7.90	410.00	66.13
	10	650.00	83.26	260.00	40.00	390.00	8.53	460.00	70.77
	11	620.00	80.26	280.00	45.16	340.00	8.71	420.00	67.74
	12	580.00	82.36	270.00	46.55	310.00	8.13	410.00	70.69
	13	570.00	87.48	300.00	52.63	270.00	8.16	410.00	71.93
	14	670.00	83.24	270.00	40.30	400.00	8.60	450.00	67.16
	15	620.00	84.20	140.00	22.58	480.00	8.38	420.00	67.74
	16	650.00	73.46	290.00	44.62	360.00	7.89	440.00	67.69
	17	650.00	72.10	280.00	43.08	370.00	7.93	470.00	72.31
	max	670.00	90.95	300.00	52.63	480.00	9.37	480.00	75.00
	mean	625.33	82.16	265.33	42.54	360.00	8.11	438.67	70.16
	SD	31.59	5.48	38.71	6.66	49.71	0.66	26.69	2.59

Participant	Trial no.	TMT (ms)	MV (cm/ms)	TMV (ms)	%TMV	DT (ms)	MA (cm)	TMA (ms)	%TMA
M05 visual occlusion	18	450.00	103.48	250.00	55.56	200.00	12.79	340.00	75.56
	4	590.00	88.65	290.00	49.15	300.00	10.86	450.00	76.27
	5	610.00	98.20	300.00	49.18	310.00	11.12	430.00	70.49
	6	550.00	101.07	290.00	52.73	260.00	12.26	420.00	76.36
	7	580.00	100.52	260.00	44.83	320.00	10.87	420.00	72.41
	8	570.00	100.45	270.00	47.37	300.00	10.77	420.00	73.68
	9	590.00	93.82	300.00	50.85	290.00	10.20	430.00	72.88
	19	450.00	106.43	230.00	51.11	220.00	12.24	340.00	75.56
	20	480.00	91.31	240.00	50.00	240.00	13.22	350.00	72.92
	12	550.00	89.91	270.00	49.09	280.00	12.84	380.00	69.09
	13	500.00	91.65	250.00	50.00	250.00	12.70	390.00	78.00
	14	550.00	81.50	270.00	49.09	280.00	12.28	400.00	72.73
	15	530.00	90.06	260.00	49.06	270.00	11.67	390.00	73.59
	16	510.00	93.52	280.00	54.90	230.00	11.68	400.00	78.43
	17	480.00	106.60	260.00	54.17	220.00	12.30	360.00	75.00
	max	610.00	106.60	300.00	55.56	320.00	13.22	450.00	78.43
	mean	532.67	95.81	268.00	50.47	264.67	11.85	394.67	74.20
	SD	52.03	7.25	21.11	2.88	36.81	0.91	34.82	2.60

Participant	Trial no.	TMT (ms)	MV (cm/ms)	TMV (ms)	%TMV	DT (ms)	MA (cm)	TMA (ms)	%TMA
M06 visual occlusion	3	600.00	75.50	300.00	50.00	300.00	6.85	390.00	65.00
	4	610.00	80.69	270.00	44.26	340.00	7.26	430.00	70.49
	5	610.00	78.07	230.00	37.71	380.00	6.79	420.00	68.85
	6	580.00	79.97	290.00	50.00	290.00	7.35	400.00	68.97
	7	570.00	93.37	270.00	47.37	300.00	6.43	450.00	78.95
	8	620.00	91.40	250.00	40.32	370.00	7.54	400.00	64.52
	9	510.00	95.23	200.00	39.22	310.00	6.99	260.00	50.98
	10	580.00	78.43	210.00	36.21	370.00	6.37	370.00	63.79
	11	530.00	93.72	230.00	43.40	300.00	6.67	350.00	66.04
	12	600.00	96.80	280.00	46.67	320.00	6.86	450.00	75.00
	13	550.00	112.67	320.00	58.18	230.00	7.87	440.00	80.00
	14	580.00	90.74	230.00	39.66	350.00	6.72	410.00	70.69
	15	590.00	85.96	260.00	44.07	330.00	7.03	440.00	74.58
	16	550.00	95.20	260.00	47.27	290.00	7.33	360.00	65.46
	17	580.00	95.42	290.00	50.00	290.00	6.93	440.00	75.86
	max	620.00	112.67	320.00	58.18	380.00	7.87	450.00	80.00
	mean	577.33	89.54	259.33	44.95	318.00	7.00	400.67	69.28
	SD	31.05	9.85	34.32	5.86	39.68	0.41	50.63	7.31

Participant	Trial no.	TMT (ms)	MV (cm/ms)	TMV (ms)	%TMV	DT (ms)	MA (cm)	TMA (ms)	%TMA
M07 visual occlusion	3	850.00	90.40	360.00	42.35	490.00	7.51	540.00	63.53
	4	840.00	81.03	330.00	39.29	510.00	7.66	520.00	61.91
	5	860.00	77.98	410.00	47.67	450.00	7.58	580.00	67.44
	6	740.00	89.87	400.00	54.05	340.00	7.70	530.00	71.62
	7	850.00	81.73	370.00	43.53	480.00	8.43	550.00	64.71
	8	860.00	84.41	240.00	27.91	620.00	8.24	510.00	59.30
	9	780.00	83.90	250.00	32.05	530.00	8.55	500.00	64.10
	10	730.00	87.06	300.00	41.10	430.00	8.46	530.00	72.60
	11	740.00	91.06	390.00	52.70	350.00	9.10	550.00	74.32
	12	870.00	83.66	400.00	45.98	470.00	8.39	590.00	67.82
	13	740.00	95.62	330.00	44.60	410.00	9.56	510.00	68.92
	14	840.00	85.84	390.00	46.43	450.00	8.47	570.00	67.86
	15	870.00	95.58	290.00	33.33	580.00	8.02	480.00	55.17
	16	820.00	91.39	400.00	48.78	420.00	7.93	570.00	69.51
	17	810.00	85.06	400.00	49.38	410.00	8.23	570.00	70.37
	max	870.00	95.62	410.00	54.05	620.00	9.56	590.00	74.32
	mean	813.33	86.97	350.67	43.28	462.67	8.26	540.00	66.61
	SD	52.87	5.22	57.50	7.53	76.95	0.56	32.29	5.20

Participant	Trial no.	TMT (ms)	MV (cm/ms)	TMV (ms)	%TMV	DT (ms)	MA (cm)	TMA (ms)	%TMA
M08 visual occlusion	3	610.00	102.96	300.00	49.18	310.00	11.56	410.00	67.21
	4	660.00	88.26	280.00	42.42	380.00	10.13	480.00	72.73
	5	700.00	88.59	280.00	40.00	420.00	8.78	540.00	77.14
	6	570.00	86.88	270.00	47.37	300.00	10.55	420.00	73.68
	7	610.00	88.45	230.00	37.71	380.00	10.72	450.00	73.77
	8	500.00	111.61	290.00	58.00	210.00	10.59	400.00	80.00
	9	680.00	104.91	260.00	38.24	420.00	9.91	410.00	60.29
	10	520.00	105.29	260.00	50.00	260.00	11.30	400.00	76.92
	11	520.00	95.63	280.00	53.85	240.00	11.45	420.00	80.77
	12	590.00	93.08	340.00	57.63	250.00	9.75	440.00	74.58
	13	520.00	103.11	270.00	51.92	250.00	9.05	370.00	71.15
	14	480.00	119.30	280.00	58.33	200.00	9.56	370.00	77.08
	15	460.00	118.59	280.00	60.87	180.00	10.55	390.00	84.78
	16	560.00	98.66	310.00	55.36	250.00	9.62	420.00	75.00
	17	520.00	91.31	260.00	50.00	260.00	9.43	380.00	73.08
	max	700.00	119.30	340.00	60.87	420.00	11.56	540.00	84.78
	mean	566.67	99.77	279.33	50.06	287.33	10.20	420.00	74.55
	SD	73.55	10.83	25.20	7.61	78.42	0.86	44.56	5.78

Participant	Trial no.	TMT (ms)	MV (cm/ms)	TMV (ms)	%TMV	DT (ms)	MA (cm)	TMA (ms)	%TMA
M09 visual occlusion	3	630.00	104.33	300.00	47.62	330.00	8.30	490.00	77.78
	4	620.00	92.74	320.00	51.61	300.00	8.98	480.00	77.42
	5	670.00	95.38	340.00	50.75	330.00	8.41	490.00	73.13
	6	610.00	96.02	270.00	44.26	340.00	8.07	440.00	72.13
	7	560.00	79.32	340.00	60.71	220.00	9.42	470.00	83.93
	8	680.00	96.25	280.00	41.18	400.00	7.26	480.00	70.59
	9	570.00	80.67	330.00	57.90	240.00	9.19	440.00	77.19
	10	670.00	73.27	330.00	49.25	340.00	8.61	500.00	74.63
	11	700.00	87.38	280.00	40.00	420.00	7.85	480.00	68.57
	12	700.00	81.71	250.00	35.71	450.00	7.81	530.00	75.71
	13	600.00	83.65	340.00	56.67	260.00	7.81	470.00	78.33
	14	580.00	82.42	310.00	53.45	270.00	7.12	470.00	81.03
	15	680.00	82.68	340.00	50.00	340.00	6.93	520.00	76.47
	16	620.00	95.15	330.00	53.23	290.00	7.19	450.00	72.58
	17	640.00	96.95	310.00	48.44	330.00	7.56	500.00	78.13
	max	700.00	104.33	340.00	60.71	450.00	9.42	530.00	83.93
	mean	635.33	88.53	311.33	49.38	324.00	8.04	480.67	75.84
	SD	46.42	8.73	29.24	6.89	64.56	0.77	26.04	4.02

Participant	Trial no.	TMT (ms)	MV (cm/ms)	TMV (ms)	%TMV	DT (ms)	MA (cm)	TMA (ms)	%TMA
M10 visual occlusion	3	810.00	85.66	500.00	61.73	310.00	7.89	660.00	81.48
	4	790.00	91.38	450.00	56.96	340.00	8.53	630.00	79.75
	5	690.00	93.07	380.00	55.07	310.00	7.90	540.00	78.26
	6	730.00	96.11	430.00	58.90	300.00	8.15	590.00	80.82
	7	760.00	84.16	440.00	57.90	320.00	7.32	620.00	81.58
	8	700.00	92.27	420.00	60.00	280.00	8.28	580.00	82.86
	9	680.00	97.81	410.00	60.29	270.00	8.47	580.00	85.29
	10	750.00	77.42	400.00	53.33	350.00	8.40	530.00	70.67
	11	640.00	86.56	350.00	54.69	290.00	7.88	490.00	76.56
	12	740.00	83.13	410.00	55.41	330.00	6.75	530.00	71.62
	13	790.00	75.87	460.00	58.23	330.00	6.93	560.00	70.89
	14	670.00	78.53	410.00	61.19	260.00	7.49	560.00	83.58
	15	790.00	77.89	430.00	54.43	360.00	7.61	630.00	79.75
	16	770.00	67.64	410.00	53.25	360.00	7.54	590.00	76.62
	17	720.00	70.24	140.00	19.44	580.00	7.47	520.00	72.22
	max	810.00	97.81	500.00	61.73	580.00	8.53	660.00	85.29
	mean	735.33	83.85	402.67	54.72	332.67	7.78	574.00	78.13
	SD	51.11	9.20	80.49	10.16	75.16	0.54	47.63	4.85

Participant	Trial no.	TMT (ms)	MV (cm/ms)	TMV (ms)	%TMV	DT (ms)	MA (cm)	TMA (ms)	%TMA
M11 visual occlusion	3	780.00	82.94	310.00	39.74	470.00	10.20	570.00	73.08
	4	680.00	94.94	300.00	44.12	380.00	10.29	450.00	66.18
	5	640.00	83.49	290.00	45.31	350.00	9.90	490.00	76.56
	6	770.00	83.98	290.00	37.66	480.00	9.57	560.00	72.73
	7	780.00	83.23	330.00	42.31	450.00	10.72	590.00	75.64
	18	650.00	89.68	290.00	44.62	360.00	10.11	510.00	78.46
	9	700.00	86.68	280.00	40.00	420.00	9.77	560.00	80.00
	10	700.00	89.33	330.00	47.14	370.00	10.78	550.00	78.57
	11	710.00	92.64	320.00	45.07	390.00	10.59	490.00	69.01
	19	590.00	97.54	280.00	47.46	310.00	11.35	480.00	81.36
	13	620.00	99.12	210.00	33.87	410.00	10.79	410.00	66.13
	14	680.00	78.91	280.00	41.18	400.00	10.51	540.00	79.41
	15	660.00	85.00	280.00	42.42	380.00	11.35	500.00	75.76
	16	660.00	99.54	310.00	46.97	350.00	11.19	530.00	80.30
	17	690.00	91.46	330.00	47.83	360.00	10.76	530.00	76.81
	mean	687.33	89.23	295.33	43.05	392.00	10.52	517.33	75.33
	SD	56.12	6.49	30.44	4.01	47.39	0.55	48.47	4.96
	max	780.00	99.54	330.00	47.83	480.00	11.35	590.00	81.36

Participant	Trial no.	TMT (ms)	MV (cm/ms)	TMV (ms)	%TMV	DT (ms)	MA (cm)	TMA (ms)	%TMA
M12 visual occlusion	3	780.00	82.94	310.00	39.74	470.00	10.20	570.00	73.08
	4	680.00	94.94	300.00	44.12	380.00	10.29	450.00	66.18
	5	640.00	83.49	290.00	45.31	350.00	9.90	490.00	76.56
	6	770.00	83.98	290.00	37.66	480.00	9.57	560.00	72.73
	7	780.00	83.23	330.00	42.31	450.00	10.72	590.00	75.64
	8	700.00	86.90	340.00	48.57	360.00	9.88	510.00	72.86
	9	700.00	86.68	280.00	40.00	420.00	9.77	560.00	80.00
	10	700.00	89.33	330.00	47.14	370.00	10.78	550.00	78.57
	11	710.00	92.64	320.00	45.07	390.00	10.59	490.00	69.01
	12	560.00	92.03	240.00	42.86	320.00	11.08	420.00	75.00
	13	620.00	99.12	210.00	33.87	410.00	10.79	410.00	66.13
	14	680.00	78.91	280.00	41.18	400.00	10.51	540.00	79.41
	15	660.00	85.00	280.00	42.42	380.00	11.35	500.00	75.76
	16	660.00	99.54	310.00	46.97	350.00	11.19	530.00	80.30
	17	690.00	91.46	330.00	47.83	360.00	10.76	530.00	76.81
	max	688.67	88.68	296.00	43.00	392.67	10.49	513.33	74.54
	mean	59.51	6.15	35.62	4.09	46.21	0.54	53.94	4.59
	SD	780.00	99.54	340.00	48.57	480.00	11.35	590.00	80.30

Participant	Trial no.	TMT (ms)	MV (cm/ms)	TMV (ms)	%TMV	DT (ms)	MA (cm)	TMA (ms)	%TMA
O01	3	660.00	72.60	360.00	54.55	300.00	4.96	460.00	69.70
visual occlusion	4	670.00	83.78	320.00	47.76	350.00	4.87	520.00	77.61
	5	820.00	65.72	420.00	51.22	400.00	4.61	630.00	76.83
(Older)	6	690.00	75.28	270.00	39.13	420.00	5.38	490.00	71.01
	7	640.00	78.33	370.00	57.81	270.00	5.46	430.00	67.19
	8	720.00	79.49	290.00	40.28	430.00	4.47	460.00	63.89
	9	660.00	68.43	290.00	43.94	370.00	4.96	500.00	75.76
	10	590.00	84.11	250.00	42.37	340.00	5.18	410.00	69.49
	11	670.00	80.56	340.00	50.75	330.00	4.78	530.00	79.10
	12	630.00	90.74	280.00	44.44	350.00	4.65	490.00	77.78
	13	610.00	80.46	250.00	40.98	360.00	5.57	340.00	55.74
	14	600.00	84.08	260.00	43.33	340.00	5.12	350.00	58.33
	15	580.00	84.79	300.00	51.72	280.00	5.29	390.00	67.24
	16	600.00	93.30	260.00	43.33	340.00	5.56	380.00	63.33
	17	690.00	79.48	270.00	39.13	420.00	4.28	530.00	76.81
	max	655.33	80.08	302.00	46.05	353.33	5.01	460.67	69.99
	mean	61.63	7.47	50.31	5.88	48.94	0.40	78.78	7.40
	SD	820.00	93.30	420.00	57.81	430.00	5.57	630.00	79.10

Participant	Trial no.	TMT (ms)	MV (cm/ms)	TMV (ms)	%TMV	DT (ms)	MA (cm)	TMA (ms)	%TMA
O02	3	730.00	70.00	340.00	46.58	390.00	6.70	550.00	75.34
visual occlusion	4	680.00	87.82	230.00	33.82	450.00	5.49	500.00	73.53
	5	700.00	80.54	270.00	38.57	430.00	5.75	560.00	80.00
	6	630.00	76.24	250.00	39.68	380.00	5.81	500.00	79.37
	7	670.00	74.37	220.00	32.84	450.00	6.17	510.00	76.12
	8	670.00	61.27	390.00	58.21	280.00	6.00	540.00	80.60
	9	710.00	79.04	300.00	42.25	410.00	6.04	590.00	83.10
	10	650.00	77.57	340.00	52.31	310.00	6.26	500.00	76.92
	11	680.00	68.24	230.00	33.82	450.00	5.71	540.00	79.41
	12	600.00	79.38	240.00	40.00	360.00	5.81	410.00	68.33
	13	530.00	86.15	230.00	43.40	300.00	6.16	420.00	79.25
	14	660.00	70.49	240.00	36.36	420.00	6.34	510.00	77.27
	15	640.00	77.63	230.00	35.94	410.00	6.26	510.00	79.69
	16	640.00	78.06	260.00	40.63	380.00	6.73	490.00	76.56
	17	600.00	71.56	200.00	33.33	400.00	6.72	490.00	81.67
	mean	652.67	75.89	264.67	40.52	388.00	6.13	508.00	77.81
	SD	50.07	6.86	53.97	7.29	54.67	0.39	47.39	3.64
	max	730.00	87.82	390.00	58.21	450.00	6.73	590.00	83.10

Participant	Trial no.	TMT (ms)	MV (cm/ms)	TMV (ms)	%TMV	DT (ms)	MA (cm)	TMA (ms)	%TMA
O03	3	500.00	90.64	290.00	58.00	210.00	9.45	400.00	80.00
visual occlusion	4	540.00	88.39	110.00	20.37	430.00	9.55	390.00	72.22
	5	530.00	83.49	230.00	43.40	300.00	9.84	310.00	58.49
	6	540.00	91.95	160.00	29.63	380.00	9.94	380.00	70.37
	7	450.00	107.64	120.00	26.67	330.00	10.78	330.00	73.33
	8	550.00	77.26	250.00	45.46	300.00	9.64	360.00	65.46
	9	500.00	84.18	310.00	62.00	190.00	9.80	390.00	78.00
	10	480.00	117.92	100.00	20.83	380.00	10.08	360.00	75.00
	11	520.00	88.78	290.00	55.77	230.00	9.38	410.00	78.85
	12	490.00	101.88	240.00	48.98	250.00	10.56	340.00	69.39
	13	480.00	91.20	190.00	39.58	290.00	9.53	340.00	70.83
	14	480.00	106.87	130.00	27.08	350.00	10.40	340.00	70.83
	15	460.00	115.35	100.00	21.74	360.00	10.46	290.00	63.04
	16	420.00	111.99	130.00	30.95	290.00	10.33	270.00	64.29
	17	480.00	117.19	130.00	27.08	350.00	10.62	310.00	64.58
	mean	494.67	98.32	185.33	37.17	309.33	10.02	348.00	70.31
	SD	36.62	13.61	76.05	14.26	68.50	0.47	41.61	6.25
	max	550.00	117.92	310.00	62.00	430.00	10.78	410.00	80.00

Participant	Trial no.	TMT (ms)	MV (cm/ms)	TMV (ms)	%TMV	DT (ms)	MA (cm)	TMA (ms)	%TMA
O04	3	830.00	77.99	360.00	43.37	470.00	7.14	550.00	66.27
visual occlusion	4	710.00	69.63	290.00	40.85	420.00	7.79	450.00	63.38
	5	760.00	87.98	250.00	32.90	510.00	8.79	510.00	67.11
	6	710.00	85.14	330.00	46.48	380.00	7.69	580.00	81.69
	7	760.00	75.17	350.00	46.05	410.00	7.92	560.00	73.68
	8	860.00	75.19	450.00	52.33	410.00	6.91	650.00	75.58
	9	860.00	82.53	330.00	38.37	530.00	7.95	590.00	68.61
	10	830.00	80.80	330.00	39.76	500.00	7.52	600.00	72.29
	11	690.00	86.76	350.00	50.73	340.00	7.90	540.00	78.26
	12	800.00	85.19	330.00	41.25	470.00	7.67	570.00	71.25
	13	830.00	80.27	350.00	42.17	480.00	7.57	680.00	81.93
	14	810.00	89.76	330.00	40.74	480.00	8.38	590.00	72.84
	15	750.00	93.03	310.00	41.33	440.00	8.24	490.00	65.33
	16	910.00	79.97	350.00	38.46	560.00	7.09	640.00	70.33
	17	820.00	83.63	330.00	40.24	490.00	7.49	530.00	64.63
	mean	795.33	82.20	336.00	42.34	459.33	7.74	568.67	71.55
	SD	63.34	6.17	42.05	4.93	58.98	0.50	61.28	5.91
	max	910.00	93.03	450.00	52.33	560.00	8.79	680.00	81.93

Participant	Trial no.	TMT (ms)	MV (cm/ms)	TMV (ms)	%TMV	DT (ms)	MA (cm)	TMA (ms)	%TMA
O05	3	700.00	85.23	330.00	47.14	370.00	6.59	520.00	74.29
visual occlusion	4	870.00	76.47	390.00	44.83	480.00	6.20	670.00	77.01
	5	670.00	83.99	310.00	46.27	360.00	6.83	420.00	62.69
	6	650.00	89.69	350.00	53.85	300.00	7.50	520.00	80.00
	7	730.00	81.77	320.00	43.84	410.00	5.83	420.00	57.53
	8	650.00	92.87	320.00	49.23	330.00	8.33	480.00	73.85
	9	760.00	81.85	310.00	40.79	450.00	8.15	530.00	69.74
	10	700.00	85.60	350.00	50.00	350.00	8.25	520.00	74.29
	11	760.00	81.37	340.00	44.74	420.00	7.67	510.00	67.11
	12	730.00	79.75	350.00	47.95	380.00	7.26	560.00	76.71
	18	880.00	78.18	340.00	38.64	540.00	6.86	590.00	67.05
	14	700.00	92.02	340.00	48.57	360.00	8.08	470.00	67.14
	15	770.00	81.70	320.00	41.56	450.00	6.89	430.00	55.84
	16	910.00	87.77	310.00	34.07	600.00	6.69	520.00	57.14
	17	860.00	79.70	290.00	33.72	570.00	6.85	520.00	60.47
	mean	756.00	83.86	331.33	44.35	424.67	7.20	512.00	68.06
	SD	86.09	4.94	24.16	5.74	89.91	0.77	65.70	7.93
	max	910.00	92.87	390.00	53.85	600.00	8.33	670.00	80.00

Participant	Trial no.	TMT (ms)	MV (cm/ms)	TMV (ms)	%TMV	DT (ms)	MA (cm)	TMA (ms)	%TMA
O06	3	680.00	87.82	340.00	50.00	340.00	9.08	480.00	70.59
visual occlusion	4	530.00	91.73	110.00	20.76	420.00	9.04	430.00	81.13
	5	530.00	88.82	110.00	20.76	420.00	9.79	370.00	69.81
	6	490.00	98.71	310.00	63.27	180.00	10.14	380.00	77.55
	7	560.00	106.14	120.00	21.43	440.00	10.09	370.00	66.07
	8	500.00	99.84	280.00	56.00	220.00	10.29	360.00	72.00
	9	550.00	98.52	110.00	20.00	440.00	11.06	430.00	78.18
	10	550.00	94.90	270.00	49.09	280.00	9.53	400.00	72.73
	11	520.00	91.75	110.00	21.15	410.00	10.58	380.00	73.08
	12	470.00	96.75	260.00	55.32	210.00	10.32	330.00	70.21
	13	450.00	128.62	270.00	60.00	180.00	10.72	340.00	75.56
	14	510.00	110.36	290.00	56.86	220.00	10.28	370.00	72.55
	15	610.00	100.90	100.00	16.39	510.00	9.92	380.00	62.30
	16	550.00	95.83	330.00	60.00	220.00	9.95	390.00	70.91
	17	490.00	119.82	120.00	24.49	370.00	10.24	350.00	71.43
	mean	532.67	100.70	208.67	39.70	324.00	10.07	384.00	72.27
	SD	57.00	11.40	96.57	18.78	112.93	0.55	38.69	4.68
	max	680.00	128.62	340.00	63.27	510.00	11.06	480.00	81.13

Participant	Trial no.	TMT (ms)	MV (cm/ms)	TMV (ms)	%TMV	DT (ms)	MA (cm)	TMA (ms)	%TMA
O07	3	1060.00	57.41	480.00	45.28	580.00	6.97	750.00	70.76
visual occlusion	4	1090.00	64.98	480.00	44.04	610.00	9.02	820.00	75.23
	5	880.00	69.67	450.00	51.14	430.00	9.57	670.00	76.14
	6	790.00	73.71	430.00	54.43	360.00	8.49	620.00	78.48
	7	1060.00	70.85	240.00	22.64	820.00	9.14	730.00	68.87
	8	870.00	68.84	420.00	48.28	450.00	9.10	630.00	72.41
	9	810.00	76.85	460.00	56.79	350.00	9.04	630.00	77.78
	10	1010.00	56.07	550.00	54.46	460.00	7.82	770.00	76.24
	11	930.00	77.29	460.00	49.46	470.00	8.06	720.00	77.42
	12	870.00	74.77	440.00	50.58	430.00	9.72	670.00	77.01
	13	1000.00	76.90	430.00	43.00	570.00	8.17	640.00	64.00
	14	780.00	79.28	420.00	53.85	360.00	8.44	590.00	75.64
	15	870.00	70.67	460.00	52.87	410.00	7.67	690.00	79.31
	16	730.00	80.16	430.00	58.90	300.00	9.30	590.00	80.82
	17	760.00	85.07	440.00	57.90	320.00	9.20	620.00	81.58
	mean	900.67	72.17	439.33	49.57	461.33	8.65	676.00	75.45
	SD	118.47	8.06	64.19	8.89	136.37	0.78	69.05	4.70
	max	1090.00	85.07	550.00	58.90	820.00	9.72	820.00	81.58

Participant	Trial no.	TMT (ms)	MV (cm/ms)	TMV (ms)	%TMV	DT (ms)	MA (cm)	TMA (ms)	%TMA
O08	3	770.00	78.73	260.00	33.77	510.00	6.53	470.00	61.04
visual occlusion	4	710.00	91.75	240.00	33.80	470.00	7.28	390.00	54.93
	5	740.00	78.79	280.00	37.84	460.00	6.42	480.00	64.87
	6	690.00	92.46	250.00	36.23	440.00	7.32	370.00	53.62
	7	570.00	96.49	200.00	35.09	370.00	7.53	380.00	66.67
	8	680.00	88.64	230.00	33.82	450.00	6.94	400.00	58.82
	9	590.00	98.67	240.00	40.68	350.00	7.34	410.00	69.49
	10	670.00	91.06	240.00	35.82	430.00	7.35	440.00	65.67
	11	600.00	102.54	240.00	40.00	360.00	6.77	400.00	66.67
	12	480.00	123.20	180.00	37.50	300.00	8.93	340.00	70.83
	13	550.00	99.53	190.00	34.55	360.00	7.59	370.00	67.27
	14	590.00	85.76	210.00	35.59	380.00	6.50	390.00	66.10
	15	590.00	81.12	230.00	38.98	360.00	7.11	410.00	69.49
	16	680.00	101.43	240.00	35.29	440.00	7.06	460.00	67.65
	17	590.00	100.40	200.00	33.90	390.00	7.06	390.00	66.10
	mean	633.33	94.04	228.67	36.19	404.67	7.18	406.67	64.62
	SD	79.25	11.41	27.48	2.31	57.05	0.61	39.76	5.19
	max	770.00	123.20	280.00	40.68	510.00	8.93	480.00	70.83

Participant	Trial no.	TMT (ms)	MV (cm/ms)	TMV (ms)	%TMV	DT (ms)	MA (cm)	TMA (ms)	%TMA
O09	3	710.00	74.91	310.00	43.66	400.00	8.40	480.00	67.61
visual occlusion	4	760.00	84.00	310.00	40.79	450.00	8.19	470.00	61.84
	5	700.00	80.79	340.00	48.57	360.00	8.99	510.00	72.86
	6	640.00	89.84	280.00	43.75	360.00	8.76	380.00	59.38
	7	640.00	80.45	330.00	51.56	310.00	7.34	460.00	71.88
	8	650.00	80.81	300.00	46.15	350.00	8.28	410.00	63.08
	9	580.00	87.25	310.00	53.45	270.00	7.62	410.00	70.69
	10	570.00	86.87	260.00	45.61	310.00	7.61	400.00	70.18
	11	650.00	84.49	280.00	43.08	370.00	7.19	440.00	67.69
	12	610.00	91.64	290.00	47.54	320.00	8.04	400.00	65.57
	13	660.00	78.18	300.00	45.46	360.00	6.78	430.00	65.15
	14	490.00	90.56	220.00	44.90	270.00	8.18	340.00	69.39
	15	570.00	76.74	290.00	50.88	280.00	7.38	420.00	73.68
	16	570.00	81.02	300.00	52.63	270.00	7.16	410.00	71.93
	17	590.00	80.69	280.00	47.46	310.00	6.66	400.00	67.80
	mean	626.00	83.22	293.33	47.03	332.67	7.77	424.00	67.91
	SD	68.01	5.12	28.70	3.75	52.44	0.70	42.73	4.23
	max	760.00	91.64	340.00	53.45	450.00	8.99	510.00	73.68

Participant	Trial no.	TMT (ms)	MV (cm/ms)	TMV (ms)	%TMV	DT (ms)	MA (cm)	TMA (ms)	%TMA
O10 visual occlusion	3	750.00	103.36	300.00	40.00	450.00	8.64	530.00	70.67
	4	730.00	102.65	280.00	38.36	450.00	8.37	470.00	64.38
	5	760.00	97.95	240.00	31.58	520.00	7.43	540.00	71.05
	6	710.00	103.84	90.00	12.68	620.00	7.23	490.00	69.01
	7	540.00	110.09	110.00	20.37	430.00	8.91	390.00	72.22
	8	730.00	103.71	120.00	16.44	610.00	8.10	490.00	67.12
	9	690.00	112.28	110.00	15.94	580.00	8.04	430.00	62.32
	10	680.00	102.02	100.00	14.71	580.00	7.57	390.00	57.35
	11	730.00	119.85	90.00	12.33	640.00	8.36	460.00	63.01
	12	640.00	99.21	250.00	39.06	390.00	7.75	420.00	65.63
	13	630.00	132.71	240.00	38.10	390.00	8.43	380.00	60.32
	14	920.00	127.40	90.00	9.78	830.00	8.19	450.00	48.91
	15	690.00	87.44	100.00	14.49	590.00	7.80	490.00	71.01
	16	740.00	126.50	90.00	12.16	650.00	8.04	420.00	56.76
	17	480.00	117.50	100.00	20.83	380.00	8.66	350.00	72.92
	mean	694.67	109.77	154.00	22.45	540.67	8.10	446.67	64.85
	SD	100.56	12.70	80.78	11.46	126.06	0.48	56.02	6.86
	max	920.00	132.71	300.00	40.00	830.00	8.91	540.00	72.92

Participant	Trial no.	TMT (ms)	MV (cm/ms)	TMV (ms)	%TMV	DT (ms)	MA (cm)	TMA (ms)	%TMA
O11 visual occlusion	3	550.00	104.38	240.00	43.64	310.00	10.53	420.00	76.36
	4	520.00	117.81	200.00	38.46	320.00	10.27	360.00	69.23
	5	540.00	122.47	210.00	38.89	330.00	10.59	350.00	64.82
	6	570.00	110.02	270.00	47.37	300.00	10.41	420.00	73.68
	7	570.00	111.73	220.00	38.60	350.00	9.55	360.00	63.16
	8	580.00	108.41	190.00	32.76	390.00	10.13	370.00	63.79
	9	500.00	110.71	200.00	40.00	300.00	9.54	350.00	70.00
	10	560.00	115.13	210.00	37.50	350.00	10.12	380.00	67.86
	11	590.00	98.33	210.00	35.59	380.00	10.21	370.00	62.71
	12	590.00	106.84	260.00	44.07	330.00	9.96	390.00	66.10
	13	530.00	115.43	210.00	39.62	320.00	10.24	330.00	62.26
	14	590.00	118.14	260.00	44.07	330.00	10.69	410.00	69.49
	15	490.00	123.16	180.00	36.74	310.00	10.15	330.00	67.35
	16	530.00	120.08	210.00	39.62	320.00	8.74	400.00	75.47
	18	450.00	146.22	170.00	37.78	280.00	10.69	300.00	66.67
	mean	544.00	115.26	216.00	39.65	328.00	10.12	369.33	67.93
	SD	41.37	11.00	29.47	3.76	29.57	0.52	34.94	4.50
	max	590.00	146.22	270.00	47.37	390.00	10.69	420.00	76.36

Participant	Trial no.	TMT (ms)	MV (cm/ms)	TMV (ms)	%TMV	DT (ms)	MA (cm)	TMA (ms)	%TMA
O12 visual occlusion	3	540.00	99.44	310.00	57.41	230.00	9.45	410.00	75.93
	4	610.00	99.59	310.00	50.82	300.00	8.52	430.00	70.49
	5	530.00	97.28	310.00	58.49	220.00	9.18	420.00	79.25
	6	610.00	95.45	300.00	49.18	310.00	9.25	420.00	68.85
	7	520.00	105.41	270.00	51.92	250.00	10.47	380.00	73.08
	8	560.00	93.02	300.00	53.57	260.00	8.96	450.00	80.36
	9	600.00	90.75	310.00	51.67	290.00	8.24	410.00	68.33
	10	610.00	86.66	310.00	50.82	300.00	8.49	450.00	73.77
	11	570.00	95.58	280.00	49.12	290.00	8.24	390.00	68.42
	12	580.00	91.30	320.00	55.17	260.00	9.78	430.00	74.14
	13	590.00	94.42	290.00	49.15	300.00	9.61	410.00	69.49
	14	580.00	95.13	300.00	51.72	280.00	9.34	420.00	72.41
	15	580.00	95.85	310.00	53.45	270.00	8.31	450.00	77.59
	16	480.00	121.46	220.00	45.83	260.00	11.30	350.00	72.92
	17	570.00	93.68	90.00	15.79	480.00	9.69	410.00	71.93
	mean	568.67	97.00	282.00	49.61	286.67	9.26	415.33	73.13
	SD	37.39	8.03	58.58	9.92	59.72	0.86	27.22	3.82
	max	610.00	121.46	320.00	58.49	480.00	11.30	450.00	80.36
